# Novel Peptide Sequences with ACE-Inhibitory and Antioxidant Activities Derived from the Heads and Bones of Hybrid Groupers (*Epinephelus lanceolatus* × *Epinephelus fuscoguttatus*)

**DOI:** 10.3390/foods11243991

**Published:** 2022-12-09

**Authors:** Pei-Teng Chan, Patricia Matanjun, Cahyo Budiman, Rossita Shapawi, Jau-Shya Lee

**Affiliations:** 1 Faculty of Food Science and Nutrition, Universiti Malaysia Sabah, Jalan UMS, Kota Kinabalu 88400, Sabah, Malaysia; 2 Biotechnology Research Institute, Universiti Malaysia Sabah, Jalan UMS, Kota Kinabalu 88400, Sabah, Malaysia; 3 Borneo Marine Research Institute, Universiti Malaysia Sabah, Jalan UMS, Kota Kinabalu 88400, Sabah, Malaysia

**Keywords:** Alcalase, ACE-inhibition kinetic, hydroxyl radical scavenging activity, reducing power, metal-chelating activity, gastrointestinal digestion

## Abstract

The heads and bones of hybrid groupers are potential precursors for angiotensin-converting enzyme (ACE)-inhibitory and antioxidant peptides. The aim of this study was to isolate the dual-action peptides from the Alcalase-treated head and bone hydrolysate of hybrid groupers followed by identification of the novel peptides. The stability of these peptides against stimulated in vitro gastrointestinal digestion (SGID) was also determined. Fraction HB-IV (less than 1 kDa) obtained from ultrafiltration showed the strongest ACE-inhibition ability (IC50: 0.28 mg/mL), which was comparable to the potency of the commercial supplement, PeptACE (IC50: 0.22 mg/mL). This fraction also demonstrated the highest hydroxyl radical scavenging and metal-chelating activities. However, further fractionation of HB-IV by a series of chromatography resulted in peptide fractions of reduced ACE-inhibitory and antioxidant activities. The hydroxyl radical scavenging and reduction potential of HB-IV were enhanced, whereas ACE-inhibitory and metal-chelating activities were reduced following SGID. A total of 145 peptide sequences were identified from HB-IV, of which 137 peptides were novel to the BIOPEP database. The results suggested that the bioactive peptides isolated from the heads and bones of hybrid groupers could be used as functional foods/ingredients with potential ACE-inhibitory and antioxidant effects.

## 1. Introduction

Bioactive peptides derived from natural sources have been receiving significant attention recently because of their various health-promoting benefits, such as antihypertensive [1,2], antioxidant [3,4], and antidiabetic effects [5]. In recent years, many therapeutic peptides have been developed within the pharmaceutical industry, with more than 50 peptide drugs on the market: approximately 170 in clinical trials, and more than 200 peptides in preclinical development [6]. They have attracted the interest of health-conscious consumers because they can be utilized as functional ingredients and nutraceuticals that can specifically contribute to health promotion and/or disease prevention in addition to providing basic nutrition [7]. Moreover, food-derived peptides are also believed to have less adverse side effects compared with synthetic compounds after long-term consumption [8].

Antihypertensive peptides are some of the most extensively studied bioactive peptides from foods because hypertension is a significant problem worldwide and among the most common risk factors for death [9]. According to a report by the World Health Organization (WHO), hypertension accounted for 9.4 million deaths worldwide in the year 2010 and this number is expected to increase as the number of adults with hypertension is predicted to rise by about 60% by the year 2025 [10,11]. In Malaysia, the national prevalence of hypertension among adults (18 years and above) was 30%, with the rate increasing with age, as reported by the National Health and Morbidity Survey in 2019 [12]. Commonly, the most effective way of treating hypertension is with synthetic drugs, such as captopril, enalapril, and lisinopril, but these drugs can cause several side effects, including cough, taste disturbance, or skin rash after long-term consumption [13]. Havelka et al. [14] reported that out of 76 hypertensive patients treated with captopril (250–388 mg/day) for two-and-a-half years, 26 patients had developed side effects (mainly cough and skin manifestations) during the first 12 months of treatment. Food-derived antihypertensive peptides, on the other hand, can effectively lower systolic (S) and diastolic (D) blood pressure (BP) without causing any noticeable adverse effects. In a study conducted by Kawasaki et al. [15], a drink containing 3 mg of peptide (Val-Tyr) was added into the daily diet of 29 hypertensive subjects for 4 weeks. After 1 week of treatment, the SBPs of these subjects were significantly reduced by 9.3 mmHg and no abnormalities in terms of body weight and urinary mineral contents (Na and K) were observed in these treated subjects.

Other than hypertension, the overproduction of free radicals, such as reactive oxygen species (ROS) and reactive nitrogen species (RNS), also pose a concern to human health because they can attack membrane lipids, protein, and DNA, which are causative factors in many diseases, including cardiovascular diseases, diabetes, and cancer [8]. Antioxidants can slow down these oxidative damages and provide protection to the body’s cells by scavenging or reducing the free radicals into less active or stable forms. Antioxidant peptides have been reported to possess various antioxidant abilities, including radical scavenging, ferric ion reducing, ferrous ion chelating, and lipid peroxidation abilities [8]. The inclusion of hydrolysates containing antioxidant peptides into the diet has led to the improvement of endogenous antioxidant enzymes (superoxide dismutase, catalase, and glutathione reductase). Tkaczewska et al. [16] reported that feeding a diet supplemented with carp skin gelatine hydrolysate (10%) to healthy rats has resulted in higher total antioxidant status and glutathione reductase levels in their blood plasma in comparison with the rats fed with a diet without hydrolysate.

Hybrid grouper, known as TGGG, a crossbreed between the giant grouper (*Epinephelus lanceolatus*) and the tiger grouper (*Epinephelus fuscoguttatus*), was the first hybrid grouper in the world, successfully produced by Borneo Marine Research Institute, Universiti Malaysia Sabah (UMS) in the year 2006 [17]. This fish gained immediate popularity from aquaculturists and seafood consumers owing to its production success and premium organoleptic properties, which led to a high commercial value [18]. Due to the growing market for the fillet of TGGG, the production of TGGG has increased tremendously from 479.03 tonnes in the year 2017 to 4401.29 tonnes in the year 2018 [19,20]. The high demand for the fillets in the food industry increases the frequency of the filleting process, and subsequently more fish waste is generated. Numerous antihypertensive and antioxidant peptides have been isolated from fish waste, such as the backbone [21], skin [22], trimmings [23], and viscera [24,25].

Although numerous antihypertensive and antioxidant fish peptides have been identified in the past, peptides from the by-products (heads and bones) of TGGG have not been reported so far. The discovery of dual-action peptides from the waste of this economically important fish would not only aid in the management of waste disposal but could also diversify the market of TGGG and enable it to fetch a higher market price. Other than to the food industry, the information gather from this study also can contribute knowledge to the scientific community including biotechnology which is interested in synthesizing the potent peptides for pharmaceutical purposes. Though the head of TGGG is used as one popular local delicacy, the fish head contains large quantities of bones with very little servable meat which still generates large amounts of waste after consumption. In contrast, using fish heads to produce biopeptides involves subjecting the whole fish head to hydrolysis with zero waste production. This is a more effective way of eliminating the waste of fish heads compared to serving it in the restaurants.

This project aimed to explore the potential of the heads and bones from TGGG as a source of novel antihypertensive and antioxidant peptides which later could be used for prevention or management of hypertension and oxidative stress. In our earlier work [26], Alcalase hydrolysis for one hour was identified as the optimal condition to obtain hydrolysate with comparatively stronger angiotensin-converting enzyme (ACE)-inhibitory and antioxidant activities. To further increase the potency of the bioactivities, purification of the Alcalase-treated head and bone hydrolysate (degree of hydrolysis of 17.7%) was conducted in this study. This was done by ultrafiltration followed by two-stages of chromatography (anion exchange chromatography and gel filtration chromatography). The sequences of the peptides in the most potent fraction were identified and their possible bioactivities were predicted by matching to the bioactive peptides database (BIOPEP). The stabilities of these peptides against in vitro gastrointestinal digestion and ACE were also assessed.

## 2. Materials and Methods

### 2.1. Sample Preparation

The hybrid groupers (TGGG) of similar weights (800–850 g) were purchased from the hatchery of Borneo Marine Research Institute, Universiti Malaysia Sabah. The fish were weighed and transported to the laboratory in ice packs on the same day. Upon arrival at the laboratory, the fish were descaled and rinsed with distilled water. After the removal of the fish fillet, fins, and viscera, the remaining matter (comprised of fish heads, backbones, rib bones, tails, and approximately 5% of flesh that was attached to the rib bones) were combined and termed as fish bones. The fish bones were frozen immediately using liquid nitrogen and followed by freeze-drying in a freeze dryer (FreeZone 12, Labconco, Kansas City, MO, USA). The freeze-dried bones were blended using a Waring blender, packed in a sealed bag, then stored at −20 °C until further use.

### 2.2. Production of Alcalase-Treated Bone Hydrolysate

The freeze-dried bones were dispersed in 0.1 M sodium phosphate buffer (pH 7.5) at the ratio of 1:50 (*w/v*). Alcalase was added into the mixture at enzyme:substrate ratio of 1:100 (*w/w*). The mixture was then incubated in a shaking incubator at 50 °C for 1 hr (Model 481, Thermo Fisher Scientific, Marietta, OH, USA). Then, the mixture was placed in a water bath (95 °C) (Isotemp GDP 20, Fisherbrand, Leicestershine, UK) for 15 min to deactivate the enzyme. The mixture was then cooled down and centrifuged at 10,000× *g* for 20 min at 4 °C (Sorvall Biofuge Primo R refrigerated centrifuge, Thermo Fisher Scientific, Waltham, MA, USA). The supernatant referred to as bone hydrolysate (Al-HB). Part of the supernatant (100 mL) was lyophilised, while the remainder was directly subjected to ultrafiltration. The freeze-dried hydrolysate was stored at −20 °C until bioactivities determination. The unhydrolysed bone hydrolysate (HB-control) was prepared in the same manner as Al-HB devoid the addition of Alcalase into the reaction mixture. Though no addition of external enzyme in the preparation of HB-control, this hydrolysate would still contain some peptides resulted from the autolysis of the proteases in the fish head and bone.

### 2.3. Determination of Protein Content and Yield of Fractions

The peptide content of lyophilised HB-control, Al-HB, and all the collected peptide fractions were determined by Lowry method using bovine serum albumin as standard [4]. A 0.5 mL of fraction (5 mg/mL) was added with 0.7 mL of alkaline-copper reagent and incubated for 20 min at room temperature. After that, 0.1 mL Folin Ciocalteu reagent (two times dilution with deionised water) was added to the mixture and allowed to incubate for 30 min at room temperature. The absorbance of the sample was measured at 750 nm and the protein content was expressed as mg/g hydrolysate. The percentage yield of Al-HB was calculated as ratio of weight of lyophilised Al-HB to the weight of lyophilised HB-control [27]. Similarly, the percentage yields of the collected fractions from each purification step were calculated as the ratio of weight of lyophilised fraction to the weight of the lyophilised unfractionated hydrolysate, i.e., Al-HB.

### 2.4. Determination of Molecular Weight Distribution of Hydrolysates Using Gel Filtration Chromatography

The molecular weight (MW) distribution of HB-control and Al-HB were determined by gel filtration chromatography on a Superdex Peptide Increase 10/300 GL size exclusion column with separation range of between 100 to 700 Da (10 × 300 mm, GE Healthcare, Chicago, IL, USA) connected to a fast protein liquid chromatography (FPLC, AKTApure, GE Healthcare, Chicago, IL, USA) equipped with a UV detector at 220 nm [28]. The HB-control and Al-HB were separately dissolved in 20 mM sodium phosphate buffer containing 0.1 M NaCl (pH 7). The mixture was filtered, and the filtrate (0.5 mL) was loaded onto the column that had been pre-equilibrated with 20 mM of sodium phosphate buffer containing 0.1 M NaCl (pH 7). The elution was performed using the same buffer at a flow rate of 0.5 mL/min. The eluted peaks were monitored at 220 nm. Carbonic anhydrous (29,000 Da), aprotinin (6512 Da), glutathione oxidised (612 Da), glutathione reduced (307 Da), and glycine (75 Da) (Sigma-Aldrich, St. Louis, MO, USA) were used as MW standards. The MW distributions of HB-control and Al-HB were estimated from the linear plot of logarithm of MW against retention time.

### 2.5. Fractionation of Alcalase-Treated Bone Hydrolysate

**Figure 1:**
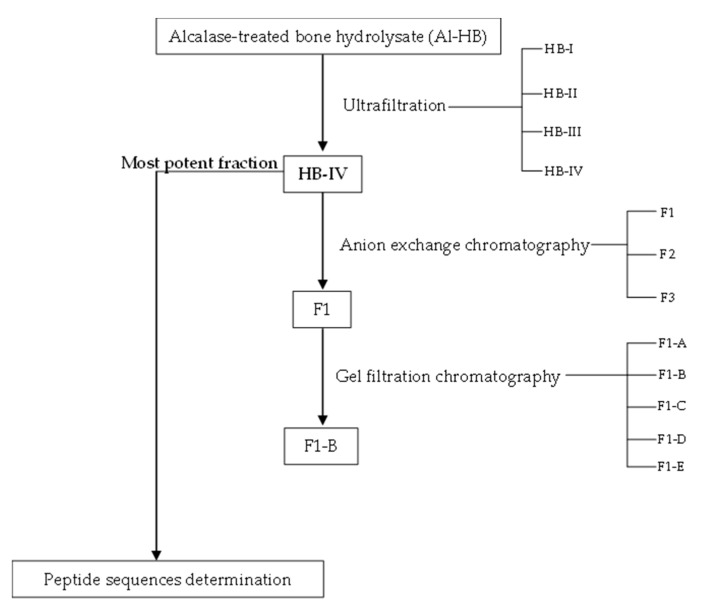
Process flow diagram for the separation of ACE-inhibitory and antioxidant peptides from the Alcalase-treated bone hydrolysate.

Figure 1 shows the flow chart for the preparation of ACE-inhibitory and antioxidant peptides from the bones of hybrid groupers. The Al-HB was fractionated in three steps, starting with ultrafiltration, then followed by anion exchange chromatography and gel filtration chromatography. After each fractionation, the fractions collected were subjected to ACE-inhibitory and antioxidant activities analyses. Fraction with the highest ACE-inhibitory and antioxidant activities was used for the next fractionation.

#### 2.5.1. Ultrafiltration

The bone hydrolysate was fractionated into four different MW fractions using a 200 mL capacity Amicon stirred cell with magnetic stirring (UFSC20001, EMD Milipore Corporation, Burlington, MA, USA) [29]. Regenerated cellulose membranes with molecular weight cut-off (MWCO) of 1, 3, and 5 kDa were used. Nitrogen gas was used to apply pressure to the stirred cell during the ultrafiltration process as indicated in the instruction manual. The hydrolysate (Al-HB) was initially subjected to ultrafiltration using a 5 kDa membrane and the retentate collected was referred as HB-I (MW > 5 kDa). The permeate then passed through the 3 kDa membrane and the resultant retentate was referred as HB-II (5 > MW > 3 kDa). Lastly, the remaining permeate was passed through a 1 kDa membrane and this time both the retentate and permeate were collected. The retentate and permeate collected were known as HB-III (3 > MW > 1 kDa) and HB-IV (MW < 1 kDa), respectively. All the fractions were lyophilised and kept at −20 °C until ACE-inhibitory and antioxidant activities determinations.

#### 2.5.2. Anion Exchange Chromatography

The most potent fraction from ultrafiltration (HB-IV) was separated by a HiTrap Q Sepharose Fast Flow (QFF) column (1.6 cm × 2.5 cm, GE Healthcare, Chicago, IL, USA) connected to a FPLC equipped with an automatic fraction collector. The HB-IV fraction was dissolved in Tris-HCl buffer (20 mM, pH 8) at a concentration of 10 mg/mL, then 5 mL of filtered sample solution was loaded onto the column that had been pre-equilibrated with the same buffer. The sample was eluted with a linear gradient of NaCl solution (0–1.0 M) in 20 mM Tris-HCl buffer (pH 8.0) at a flow rate of 1.5 mL/min. The elution was observed at 220 nm and the peak fractions were collected at a volume of 1 mL. The fractions collected were concentrated by lyophilisation and were analysed for ACE-inhibitory and antioxidant activities. The fraction showing the strongest bioactivities were subjected to further separation by gel filtration chromatography.

#### 2.5.3. Gel Filtration Chromatography

The most potent fraction from anion exchange chromatography (F1) was fractionated using FPLC connected to a Superdex 30 Increase 10/300 GL [30]. The F1-A fraction was solubilised in 20 mM sodium phosphate buffer containing 0.1 M NaCl (pH 7) (80 mg/mL). The column was pre-equilibrated with 20 mM sodium phosphate buffer with 0.1 M NaCl (pH 7), then 0.5 mL of filtered sample solution was injected into the column. The elution was conducted in the same buffer using an isocratic flow rate of 0.5 mL/min and was monitored at 220 nm. Each peak fraction collected at a volume of 0.5 mL was pooled, concentrated by lyophilisation, and used to analyse the ACE-inhibitory and antioxidant activities. Five MW standards including carbonic anhydrous (29,000 Da), aprotinin (6512 Da), glutathione oxidised (612 Da), glutathione reduced (307 Da), and glycine (75 Da) were run through the column using the same conditions and were used for MW calculations. The MW of the fractions were estimated from a linear plot of logarithm MW against retention time of the standards.

### 2.6. Determination of Amino Acids Composition of Fractions

The amino acids composition of the peptide fractions was determined following the method described by Rutherfurd and Gilani [31]. In brief, the peptide fractions were hydrolysed with 6 N HCl at 110 °C for 24 hr. The tryptophan content was determined after alkaline hydrolysis. Amino acid analysis was conducted on a HPLC system (Agilent 1200 series, Agilent Technologies, Santa Clara, CA, USA) fitted with a Zorbax Eclipse-AAA column (4.6 × 150 mm, 3.5 μm, Agilent Technologies, Santa Clara, CA, USA) after online precolumn derivatization with o-phthalaldehyde (OPA) and 9-fluorenylmethyl chloroformate (FMOC). The derivatized sample (18 μL) was then injected into the column set at 40 °C. The sample was eluted at flow rate of 2.0 mL/min using 40 mM sodium phosphate buffer (pH 7.8) (eluent A) and acetonitrile:methanol:water (45:45:10, *v/v/v*) (eluent B) following a gradient elution of 0% B in 0–18.1 min, 57% B in 18.1–18.6 min, 100% B in 18.6–23.2 min, and 0% B in 23.2–26 min. The separation was observed with both FLD and DAD detectors. The excitation and emission wavelength of FLD were set at 340 and 450 nm, respectively, from time 0 to 15 min, then switched to 266 and 305 nm, respectively, until the end of analysis (26 min). The absorbance for the DAD detector was set at 338 nm and 262 nm throughout the elution. The AA were identified and quantified by comparing with the retention times and peak areas of standards (Agilent Technologies, Santa Clara, CA, USA). The amino acids were identified and quantified by comparing with the retention times and peak areas of standards (Agilent Technologies, Santa Clara, CA, USA). The results were presented as percentage total amino acids (%).

### 2.7. Determination of ACE-Inhibitory Activity of Fractions

The ACE inhibitory activity of peptide fractions was measured according to the method described previously [32,33]. Briefly, the fraction of various concentration was mixed with HHL (8.3 mM) and pre-incubated at 37 °C for 5 min. Then, freshly prepared ACE (25 mU/mL) was added, and the reaction mixture was incubated at 37 °C for 60 min. After incubation, the reaction was stopped with HCl (1 N) and the hippuric acid was extracted using ethyl acetate, then evaporated into dryness in a vacuum oven. Distilled water was then added to dissolve the hippuric acid, and the absorbance was read at 228 nm using UV-Vis spectrophotometer (Lambda 35, Perkin Elmer, Waltham, MA, USA). The ACE-inhibitory activity was calculated using the following Equation (1):

ACE-inhibitory activity (%) = [(A − B)/(A − C)] × 100%
(1)

where A is the absorbance of control, B is the absorbance of the reaction mixture, and C is the absorbance of mixture when the stop reaction solution was added before the reaction occurred. The results were represented in IC50 (mg/mL), i.e., the concentration of fraction needed to inhibit 50% of ACE activity, which was determined by linear regression analysis of ACE-inhibitory activity (%) versus fraction concentration. The IC50 value of PeptACE, a commercial antihypertensive supplement which is composed of a mixture of nine small peptides derived from bonito fish (http://us.naturalfactors.com/ assessed on 7 September 2017) was also determined for comparative purposes.

### 2.8. Determination of In Vitro Antioxidant Activities of Peptide Fractions

#### 2.8.1. Hydroxyl Radical Scavenging Activity

Hydroxyl radicals were generated by Fenton reaction [34]. A reaction mixture containing 0.1 mL ferrous sulphate (FeSO_4_) (10 mM), 0.1 mL ethylenediaminetetraacetic acid (EDTA) (10 mM), 0.5 mL 2-deoxyribose (10 mM), and 0.9 mL sodium phosphate buffer (0.1 M, pH 7.4) was mixed with 0.2 mL of sample. The reaction was then initiated by the addition of 0.2 mL of hydrogen peroxide (H_2_O_2_) (10 mM). The mixture was then incubated at 37 °C for 90 min. Following that, the reaction was stopped by adding 1 mL of cold trichloroacetic acid (TCA) (2%) and 1 mL of thiobarbituric acid (TBA) (1%). The mixture was then heated in a boiling water bath for 15 min. The mixture was cooled to room temperature, and the absorbance of the resulting pink colour in the samples was measured at 532 nm using a UV-Vis spectrophotometer. The hydroxyl radical scavenging activity (%) is calculated according to the following Equation (2):

Hydroxyl radical scavenging activity (%) = (A_control_ − A_sample_)/(A_control_) × 100% (2)

where A_control_ is the absorbance of reaction mixture without the hydrolysate and A_sample_ is the absorbance of reaction mixture with hydrolysate. The radical scavenging activity of peptide fractions were presented as EC50 (mg/mL), which was defined as the concentration of fraction needed to scavenge 50% of the hydroxyl radical activity. The EC50 value was determined by linear regression analysis of hydroxyl radical scavenging activity (%) versus sample concentration.

#### 2.8.2. Reducing Power

The reducing power of the peptide fractions was measured using the method described by Najafian and Babji [4]. A 0.2 mL of sample aliquot (1 mg/mL) was added to 0.5 mL of sodium phosphate buffer (0.2 M, pH 6.6) and 0.5 mL of potassium ferricyanide solution (1%). After an incubation at 50 °C for 20 min, 0.5 mL of TCA (10%) was mixed with the reaction mixture, and the reaction mixture was centrifuged at 3500× *g* for 10 min at 4 °C. Finally, 0.5 mL of the supernatant solution was subsequently added to 0.5 mL of distilled water and 0.1 mL ferric chloride solution (0.1%). After 2 min, the absorbance was measured at 700 nm against blank using UV-Vis spectrophotometer. An equivalent of water instead of the hydrolysate was used as blank. The results were presented as absorbance unit at 700 nm. Higher absorbance of reaction mixture indicated a higher the reducing power.

#### 2.8.3. Metal-Chelating Activity

The metal-chelating activity of fractions was measured using the method of Najafian and Babji [4]. Fraction (0.25 mL) was added to 0.925 mL distilled water and 25 μL ferrous chloride (2 mM). The reaction was initiated with the addition of ferrozine (50 μL, 5 mM), followed by incubation at room temperature for 20 min. The absorbance of the mixture was then measured at 562 nm. Distilled water was used for the control instead of fraction. The metal-chelating activity (%) is calculated according to the following Equation (3):

Metal-chelating activity (%) = [1 − (A/B)] × 100%
(3)

where A is the absorbance of fraction and B is the absorbance of the control. The results were presented as EC50 (mg/mL), i.e., concentration of fraction needed to cause 50% decrease of chelating activity. The EC50 value was determined by linear regression analysis of metal-chelating activity (%) versus sample concentration. Ethylenediaminetetraacetic acid (EDTA) was used as a positive control.

### 2.9. Identification of Peptide Sequences

After investigating the potency of the purified peptide fractions, the peptide sequence of the most potent fraction was identified. Fraction was initially subjected to desalting procedure using the Pierce desalting peptide spin column (Thermo Fisher, Marietta, OH, USA). The separation of peptides was performed using AdvanceBio Peptide Map, C18 column (2.1 × 100 mm, 2.7 µM, Agilent Technologies, Santa Clara, CA, USA) at flow rate of 15 µL/min [35]. The mobile phases used were: (A) deionised water containing 0.1% formic acid, and (B) acetonitrile containing 0.1% (*v/v*) formic acid. The gradient of the HPLC system was as follows: (a) 0–5 min, 10% B; (b) 5–115 min, 10–95% B; (c) 115–120 min, 95% B; (d) 120–135 min, 95–100% B, and (e) 140–150 min, 10% B. The eluent was analysed using the electrospray ionisation-quadrupole-time-of flight system (ESI-QTOF, model 6520, Agilent Technologies, Santa Clara, CA, USA) with the following condition: (a) mass range: 100–2000 m/z; (b) collision energy: 6 V/100 Da (offset −2); (c) flow rate: 15 µL/min; (d) ion spray sources: 3.5 kV; (e) drying gas: nitrogen at temperature of 350 °C with flow rate of 10 L/min; (f) nebuliser pressure: 3 psig; (g) fragmentor voltage: 110 V; and (h) fragmentation mode: collision induced dissociation (CID). Peak studio version 6.0 was used for the de novo sequencing. The identified peptides were subjected to in silico analysis using the bioactive peptide database (BIOPEP), a tool used to identify potential bioactive fragments within identified peptides [36]. PeptideRanker (https://bioware.ucd.ie/compass/biowareweb/ accessed on 9 July 2021) was also used to predict the probability of the peptides being bioactive based on the training set from available bioactive peptide databases, which includes but is not limited to the antioxidant and ACE-inhibitory peptides [37]. The maximum score represents the most active peptides, and lowest scores denote the least active peptides.

### 2.10. Stability of Peptide Fraction against In Vitro Stimulated Gastrointestinal Digestion

The in vitro stimulated gastrointestinal digestion (SGID) was conducted according to the method described by Toopcham et al. [30] with slight modification. The stimulated saliva, gastric, and intestinal solution were prepared as follows: saliva solution (0.24 g of disodium phosphate (Na_2_HPO_4_), 0.02 g of potassium dihydrogen phosphate (KH_2_PO_4_), and 0.8 g of NaCl dissolved in 100 mL of distilled water, pH 6.8); gastric solution (HCl solution (5 N) containing 0.03 M of NaCl, pH 1.2); intestinal solution (0.3 g of bile salt in 35 mL of sodium bicarbonate (NaHCO_3_, 0.1 M) (pH 8)).

Sample (0.5 g) was added with saliva solution (5 mL) and the mixture was mixed using a stomacher for 1 min followed by being shaken at 37 °C for 2 min in a shaking incubator. After that, the mixture was adjusted to pH 2 using 5 N HCl. Gastric solution (15 mL) and pepsin (1%, *w/w*) was then added to the mixture and incubated at 37 °C for 120 min with continuous shaking at 90 rpm in a shaking incubator. After 120 min, the mixture was neutralised to pH 7 using 5 M sodium hydroxide (NaOH). One of the test tubes was centrifuged at 5000× *g* for 15 min at 4 °C. The supernatant collected referred to as pepsin-digest was lyophilised. After that, 15 mL of intestinal solution, 1% of pancreatin (*w/w*), 5 mL of NaCl (120 mM), and 5 mL of KCl (5 mM) were added to the remaining centrifuge tube. The mixture continued to incubate at 37 °C for another 120 min. After 240 min, the centrifuge tube was heated in a 98 °C water bath for 15 min to deactivate the enzymes. After cooling down, the mixture was centrifuged at 5000× *g* for 15 min at 4 °C. The supernatant collected referred to as pancreatin-digest was lyophilised. Both pepsin- and pancreatin-digests were stored at −20 °C until used for ACE-inhibitory and antioxidant activities determination.

### 2.11. Stability of Peptide Fraction against ACE Using Pre-Incubation Experiment

The stability of HB-IV and PeptACE against ACE was determined by following the method described by Cinq-Mars et al. [38]. For pre-incubation with ACE, HB-IV, and PeptACE in 50 μL of sodium borate buffer (50 mM, pH 8.3 mM) was incubated with 50 μL of ACE (25 mU/mL) for 1 h at 37 °C. The pre-incubation activity was terminated by heating the mixture at 95 °C for 5 min. Then, 150 μL of HHL (8.3 mM) was added to initiate the reaction and a fresh aliquot of ACE (50 μL) was added. The reaction mixture then was incubated at 37 °C for 60 min. The reaction was stopped by adding 250 μL of 1 N HCl. The ACE-inhibitory activity was then calculated following the equation mentioned in Section 2.7 and the results were presented as IC50 (mg/mL). The IC50 value obtained was then compared to the without pre-incubation to determine the type of ACE-inhibition.

### 2.12. Determination of ACE-Inhibition Kinetics

The inhibitory kinetics of HB-IV and PeptACE on ACE were determined according to the method of Ghassem et al. [39]. The ACE-inhibition were measured by varying the concentration of HHL (0.5, 1, 2, 4, and 6 mM) in the absence and presence of various concentrations of HB-IV and PeptACE. The data obtained were used to produce Lineweaver-Burk plots of 1/initial velocity over incubation time (1/mM/min) versus HHL concentration (1/mM). The maximum velocity (Vmax) and Michaelis constant (Km) were determined from the Lineweaver-Burk plots and the inhibition type can be identified by comparing the Vmax and Km values to those in the absence of sample.

### 2.13. Statistical Analysis

All the experiments were conducted in triplicate and the data were presented as mean ± standard deviation (SD). A one-way analysis of variance (ANOVA) followed by Tukey’s multiple range test were used to compare the means of bioactivities of different fractions. All the statistical analyses were conducted using SPSS program version 25. Differences between means were considered significant at *p* < 0.05.

## 3. Results and Discussion

### 3.1. ACE-Inhibitory and Antioxidant Activities of Alcalase Hydrolysate

**Figure 2:**
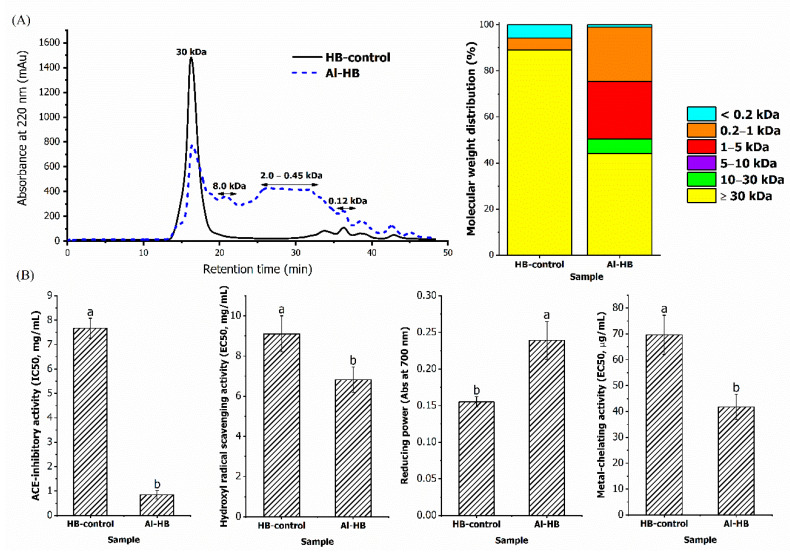
(**A**) The molecular weight distribution of HB-control and Al-HB after passage through a Superdex Peptide 10/300 GL column. (**B**) The ACE-inhibitory and antioxidant activities of the HB-control and Al-HB. The values represent the mean ± SD. Means without a common letter (a, b) on the bars indicate significant difference at *p* < 0.05.

The MW profiles of HB-control (unhydrolysed) and Al-HB are shown in Figure 2A. The protein in the bones of TGGG was cleaved by Alcalase hydrolysis resulted in the production of peptides with varying MWs. Prior to enzymatic hydrolysis, HB-control consisted of approximately 86% of peptides with MW of 30 kDa. After Alcalase hydrolysis, the height of the major peak was reduced by 50% and more peaks with MW below 30 kDa were seen eluted after 20 min. The majority of the peptides in Al-HB (49%) had MW ranging from 0.45 to 2.0 kDa, and approximately 6% was in the MW of 8 kDa, and the remaining were made up of peptides with MW below 0.20 kDa. The protein content of Al-HB (114.78 mg/g) was higher than HB-control (42.60 mg/g), higher soluble protein was most likely due to the reduction of secondary structure and generation of peptides with low molecular weight via hydrolysis [4].

The ACE-inhibitory and antioxidant potential of Al-HB was found stronger than the HB-control (Figure 2B) because a considerable number of peptides were released from the bones of TGGG through Alcalase hydrolysis as shown in Figure 2A. This confirms that peptides need to be released from the parent protein in order to display higher bioactivities. The ACE-inhibition potential of Al-HB was higher than the control; with its IC50 value (0.85 mg/mL) nine times lower than HB-control (7.67 mg/mL) (*p* < 0.05). The IC50 of Al-HB is found to be lower than those hydrolysates from various fish sources, such as chum salmon skin (IC50: 1.08 mg/mL) [40] and skate skin (IC50: 1.89 mg/mL) [33], but is higher than values reported for snakehead fish (IC50: 0.038 mg/mL) [41] and tilapia (IC50: 0.54 mg/mL) [30]. Other than ACE-inhibitory activity, the Al-HB also displayed antioxidant potential with the ability to scavenge hydroxyl radicals, to reduce ferric ion to ferrous ion, and to chelate ferrous ion. The Al-HB recorded a higher hydroxyl radical scavenging ability than control with a lower EC50 value of 6.82 mg/mL (*p* < 0.05). This EC50 value is lower than those reported in abalone muscle (EC50: 10.77 mg/mL), and scallop muscle (EC50: 13.76 mg/mL) [42]; but higher than Bluefin leatherjacket skin (EC50: 1.15 mg/mL) [43]. The ferrous ion chelating activity of Al-HB, with the EC50 value of 41.76 µg/mL is also found to be lower than patin sarcoplasmic protein (EC50: 0.77 mg/mL) [4], abalone (EC50: 0.48 mg/mL), and scallop muscle (EC50: 0.40 mg/mL) [42].

### 3.2. Purification of ACE-Inhibitory and Antioxidant Peptides

#### 3.2.1. Ultrafiltration

After ultrafiltration, Al-HB was separated into four fractions with different MW, i.e., >5 kDa (HB-I), 3–5 kDa (HB-II), 1–3 kDa (HB-III), and <1 kDa (HB-IV). The yield of these four fractions were in the range of 14–25% with the HB-IV fraction with the lowest yield (Table 1). The protein contents ranged between 37.41 mg/g and 104.34 mg/g, which increased with MW. Ultrafiltration of the hydrolysates produced fractions with increased ACE-inhibitory activity except for the fraction above 5 kDa (HB-I), whereby the IC50 showed no significant difference (*p* > 0.05) as compared to Al-HB (Table 1). The IC50 values of HB-II, HB-III, and HB-IV fractions were in the range of 0.28–0.44 mg/mL (*p* > 0.05), with HB-IV fraction showing the lowest value (0.28 mg/mL). The IC50 value of HB-IV fraction was three times lower than that of Al-HB and was comparable to the commercially available antihypertensive supplement, PeptACE (IC50: 0.22 mg/mL). The ACE-inhibition of HB-IV is higher than those reported previously on the peptide with similar MW from Alaska Pollack frame (IC50: 0.46 mg/mL) [44] and chicken thigh skin (IC50: 0.42 mg/mL) [45]. The current results are in corroboration with reported results that comparatively shorter chain length peptides were the main contributors for the ACE-inhibitory activity because when peptides with MW above 5 kDa were removed through ultrafiltration, the ACE-inhibition was greatly improved. Small size peptides are more efficient because they were able to fit into the active site of ACE as opposed to larger polypeptides where their access was blocked by the N-terminal ‘lid’ at the active site of ACE [46].

Other than peptides size, the ACE-inhibitory was also regulated by the amino acids composition (Table 2). The presence of proline and negatively charged amino acids (NCAA) are known to contribute positively to the ACE-inhibition via hydrophobic interaction and metal chelation [47]. The presence of proline (1.53–4.84%) and a significantly high amount of NCAA (23.46–27.57%) in HB-II, HB-III, HB-IV, and PeptACE, caused noticeably higher readings in the ACE-inhibitory capacity. However, the high proline content (10.07%) in the HB-I fraction did not contribute positively to the ACE-inhibitory activity. This may be due to the low composition of NCAA in this fraction (20.94%) which in turn reduces the ionic interaction in the ACE active site. This is in accordance with the findings reported by Zhang et al. [47], whereby the proper modulation of proline and NCAA at a moderate percentage can promote a strong ACE-inhibitory activity.

All the ultrafiltrated fractions (HB-I, HB-II, and HB-III) exhibited similar hydroxyl radical scavenging activity like Al-HB, except for HB-IV fraction (MW < 1 kDa), which exhibited two lower EC50 (3.48 mg/mL vs. 6.97 mg/mL). This showed that the peptides with MW below 1 kDa can demonstrate a stronger hydroxyl radical scavenging effect. However, all the ultrafiltrated fractions were found to have much lower hydroxyl radicals scavenging ability than PeptACE (EC50: 0.44 mg/mL) (*p* < 0.05). The hydroxyl radicals scavenging activity of the HB-IV fraction was comparable to the previously reported data on the peptide fraction (MW < 1 kDa) from monkfish (EC50: 4.20 mg/mL) [48]. Similarly, the HB-IV fraction also exhibited the highest metal-chelating activity among the ultrafiltrated fractions (*p* < 0.05). Its chelating ability was higher than similar fractions from the other hydrolysates derived from tuna [28] and mushroom [49]. Previous studies on the on the skin of bluefin leatherjacket [43] and muscle of monkfish [48] also disclosed that peptides with MW below 1 kDa displayed higher antioxidant potential, in agreement with the present study. The high hydroxyl radicals and metal-chelating activity exhibited by HB-IV fraction may be attributed by the high content of aromatic amino acids (AroAA) (methionine, phenylalanine, and tyrosine) (9.28%) and hydrophobic amino acids (HAA) (44.28%) (*p* < 0.05). Pownall et al. [50] reported a strong positive correlation between the hydroxyl radicals scavenging and metal-chelating activities with the amount of AroAA and HAA in pea seed hydrolysate. Udenigwe and Aluko [51] suggested that the aromatic amino acids can use its excess electrons to scavenge free radicals and terminate the oxidative chain reactions of the radicals.

Unlike hydroxyl radical scavenging and metal-chelating activity, the effect of peptide size on reducing potential showed a different trend, in which the reducing power decreased as the MW of peptides decreased. Among the ultrafiltrated fractions, the HB-I fraction (MW > 5 kDa) exhibited the strongest reducing power (0.352) (*p* < 0.05) and was comparable to PeptACE (0.336) (*p* > 0.05). From the literature, long-chained peptides were reported to be more potent than short-chained peptides in term of reducing power because of the additive and synergistic effect of reducing groups in long chained peptides. For example, a study on hemp seed hydrolysate showed that peptides with MW between 5 to 10 kDa have approximately a 50% higher reducing capacity than peptides with MW below 1 kDa [52].

The results of current study show that membrane ultrafiltration brought about peptide fractions with increased potency against ACE, hydroxyl radicals, and ferrous ions. Ultrafiltration could isolate and concentrate the potent peptides with lower MW, giving rise to fractions with higher bioactivities. There are studies however that have contradicted to the current findings, Raghavan and Kristinsson [29] found that the ACE-inhibition of tilapia hydrolysate was higher than its ultrafiltrated fractions (>30 kDa, 10–20 kDa, and <10 kDa). As for antioxidant activities, Saidi et al. [28] reported that ultrafiltration decreased the metal-chelating properties of tuna hydrolysate rather than increasing the activity. In another study on hoki frame [53], peptides with MW between 1 to 3 kDa showed higher hydroxyl radical scavenging activity than peptides with MW below 1 kDa. The discrepancies in the results show that bioactivities of peptides are not only affected by the size of peptides. Other factors, such as the sequences of peptides in the fraction, also play a role in affecting the bioactivities.

Current findings show that peptides with MW below 1 kDa (HB-IV) were the main contributor to the ACE-inhibitory, hydroxyl radical and metal-chelating activities while peptides with MW above 5 kDa (HB-I) possessed greater reducing power potency. It is fascinating to disclose that the ACE-inhibitory potential of HB-IV fraction is as good as the commercial ACE-targeting biopeptides. In addition to the exhibited multiple biological functions (ACE-inhibitory, hydroxyl radical scavenging, and metal-chelating activities) with higher activity, the size of peptides in HB-IV was also one of the reasons this fraction was selected for further fractionation with anion exchange chromatography. This was because the smaller size peptides can pass through the intestinal barrier and be absorbed more easily than larger size peptides [54].

#### 3.2.2. Anion Exchange Chromatography

**Figure 3:**
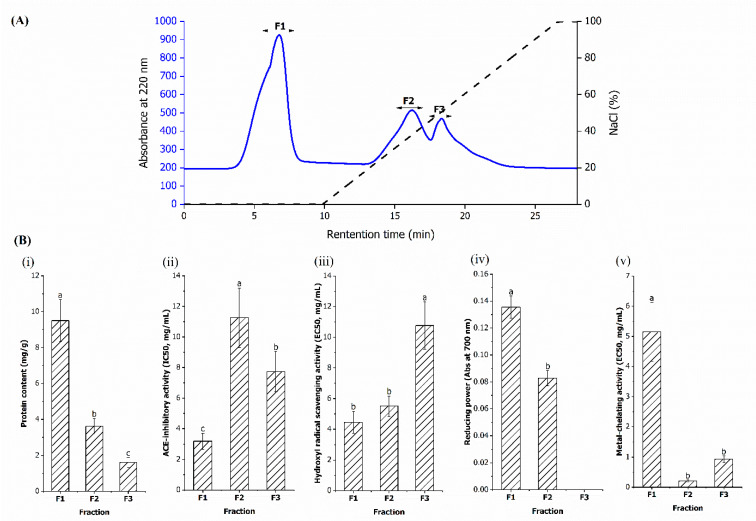
(**A**) The elution profile of HB-IV fraction separated by anion exchange chromatography on a QFF column. (**B**) The protein content and bioactivities of each collected fractions (F1–F3). The values represent the mean ± SD. Mean without a common letter (a–c) on the bars indicate significant difference at *p* < 0.05.

As shown in Figure 3A, three fractions, namely F1, F2, and F3 fractions, were separated from HB-IV fraction using an anion exchange column. Among them, F1 was the unbound fraction eluted by 20 mM Tris-HCl (pH 8), while F2 and F3 were the bound fractions eluted by the same buffer with linear increasing of NaCl concentration. This indicated that the F1 fraction contained peptides that have net positive or neutral charge at pH 8, while F2 and F3 were net negative charged peptides with the latter fraction having higher anionic strength. The protein contents of the fractions ranged between 9.51 mg/g to 1.61 mg/g with F1 fraction has the highest and F3 fraction has the lowest protein content. Similarly, F1 fraction has the highest yield of 4.33%, followed by F2 (3.10%) and F3 fraction (1.97%).

All the anion exchange fractions exhibited different degrees of ACE-inhibitory and antioxidant activities (Figure 3B) with the F1 fraction exhibiting the highest bioactivities (lowest IC50 and EC50 values) in term of ACE-inhibitory (IC50: 3.18 mg/mL), hydroxyl radical scavenging (EC40: 4.45 mg/mL) and reducing power (0.136) compared to the F2 and F3 fractions (*p* < 0.05). The IC50 values of F1 fraction was two to three times lower than the F2 and F3 fractions. The higher ACE-inhibitory activity reported in the F1 fraction might be related to the presence of significantly higher PCAA, such as arginine (13.25%) and histidine (5.90%) (Table 3). It was postulated that the positive charge of the guanidine group from arginine contribute to ACE-inhibition via binding to an anionic binding site of ACE that is away from the active site [46,55]. This is supported by Quirós et al. [56] who found that peptide sequences with arginine exhibited two times higher ACE-inhibitory activity than peptide sequences consisting of proline or leucine. Apart from the composition, the position of PCAA in peptide sequences also affects the ACE-inhibitory capacity. Previous structural-activity studies suggested that strong ACE-inhibitory activity was observed when PCAA was located at the C-terminal of peptides [57,58]. It is worth noting that though the F1 fraction had lower PCAA than F3 fraction, it still exhibited higher ACE-inhibitory activity (*p* < 0.05). This suggests that F1 fraction may have more PCAA at the C-terminal and therefore able to exhibit a better inhibitory effect. Additionally, the high HAA (42.74%) (*p* < 0.05) in the F1 fraction may be another reason behind its higher ACE-inhibitory potential than the F2 and F3 fractions since ACE also prefer peptides containing HAA [59].

Other than ACE-inhibitory activity, the hydroxyl radical and reducing power exhibited by the F1 fraction may be contributed to by amino acids containing imidazole and sulfhydryl groups with proton donating ability, such as histidine (5.90%) and methionine (1.39%) [60]. It is worth noting that histidine was only found in the F1 fraction and thus brought about higher hydroxyl radical scavenging activity, deduced from its lower EC50 value than F2 and F3 fractions. In contrast to hydroxyl radical scavenging activity and reducing power, higher metal-chelating activity was observed in F2 (EC50: 0.21 mg/mL) and F3 fractions (EC50: 0.93 mg/mL) (*p* < 0.05). The high metal-chelating activity may be attributed to the high amount of negatively charged amino acids (NCAA) found in these fractions (*p* < 0.05). The carboxyl groups in NCAA were more easily attracted to the positively charged ferrous ion for chelation [61]. This finding is similar with Lin et al. [62], who reported that the increase in glutamic acid could lead to better metal-chelating activity in hairtail fish.

Based on the ionic characteristics, the peptides with basic properties exhibited better bioactivities in ACE-inhibitory, hydroxyl radicals, and reducing power, while the peptides with acidic properties had stronger metal-chelating ability. The same findings were noted for peptides isolated from tilapia fillet [30] and Alaska Pollack frame [44]. It is noted that not all past research findings agree with the current findings; studies on bigeye tuna [63], stingray cartilage [64], and fermented goat milk [65] reported higher ACE-inhibitory and antioxidant activities in acidic fractions than basic fractions. Another study reported that both acidic and basic fractions derived from casein exhibited equally good antioxidant potential [66]. This shows that the effect of ion properties on bioactivities is sample specific. From the present findings, the F1 fraction demonstrated higher capacity in multiple bioactivities, i.e., ACE-inhibitory, hydroxyl radical scavenging, and reducing power making it a better candidate for the next step of fractionation using gel filtration chromatography.

#### 3.2.3. Gel Filtration Chromatography

**Figure 4:**
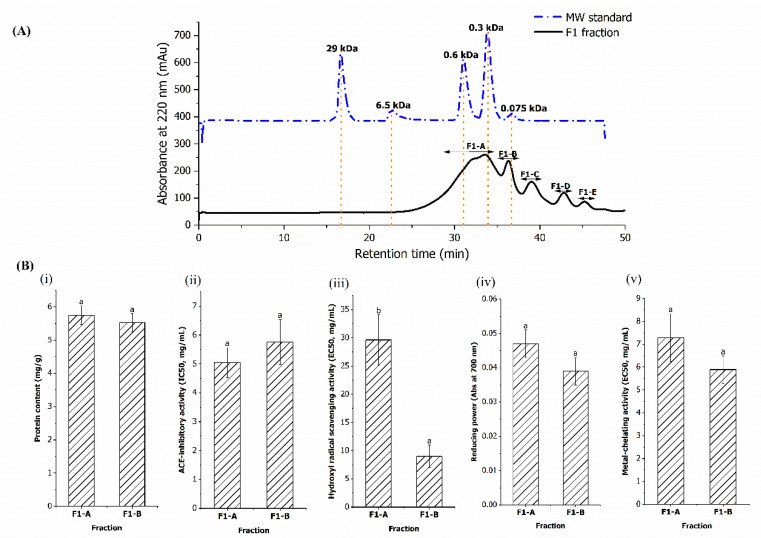
(**A**) The elution profile of F1 fraction on a Superdex Peptide 10/300 GL gel filtration column. (**B**) The protein content and bioactivities of the collected fractions (F1-A and F1-B). The values represent the mean ± SD. Means without a common letter (a, b) on the bars indicate significant difference at *p* < 0.05.

Gel filtration is a method that separates substances based on differences in molecular dimensions and at the same time desalts the protein solution [4]. The F1 fraction obtained from anion exchange chromatography was loaded into a gel filtration column and five peaks, defined as F1-A, F1-B, F1-C, F1-D, and F1-E, were obtained (Figure 4A). According to Figure 4A, the F1-A fraction was the major peak, estimated to contain over 60% of peptides with the average MW ranged between 200 to 660 Da, while F1-B consisted of 16.80% of peptides with average MW ranged between 75 to 200 Da. The average MW of the remaining three fractions (F1-C to F1-E) were smaller than glycine, which was the smallest amino acid with MW of 75 Da [67]. This indicates that these three fractions did not contain peptides or amino acids and the detected peaks were most probably consisted of other organic materials that absorbed at 220 nm [68].

ACE-inhibitory and antioxidant activities were only detected in the F1-A and F1-B fractions, but not in the other three fractions (Figure 4B). The bioactivities of F1-A and F1-B were similar (*p* > 0.05), except for hydroxyl radical scavenging activity, in which F1-B recorded significantly lower EC50 value (9.01 mg/mL) (*p* < 0.05), indicating that the peptides in F1-B are more potent than F1-A. The high potency peptides in F1-B most probably consisted of dipeptides and/or free amino acids with MW below 200 Da. Previous findings also found that dipeptides with MW ranged between 172 to 204 Da exhibited high ACE-inhibitory activity in salmon [23] and Alaska pollack [58]. Study by Weng et al. [69] also found that free amino acids, such as arginine and tyrosine derived from the blue shark skin gelatine, exhibited higher hydroxyl radicals scavenging activity than the dipeptides and tripeptides. The present results shows that the F1-B fraction displayed better bioactivities than the F1-A fraction, but the low yield of the F1-B fraction (0.87%) makes this fraction economically impractical to be used as a functional ingredient.

Numerous studies had reported that fractionation on hydrolysate using sequential purification steps would result in peptide fractions with higher potency [4,21,70]. However, current findings show that purification processes after ultrafiltration caused a decrease in the ACE-inhibitory and antioxidant activities of the fractions (Table 4), which was similar to the reports on shark meat [71] and velvet beans [72]. After purification, the bioactivities of the F1-B fraction particularly ACE-inhibitory and metal-chelating capacities were more than 20 times lower than the HB-IV fraction. The reduction of bioactivities after ultrafiltration showed that the bioactivities in HB-IV were attributed to the combined effect of the peptides. Further fractionation separated these bioactive peptides and resulted in weaker activities. Due to the low bioactivities of the F1-B fraction, the sequences of its peptides were not of interest and thus were not subjected to identification. The peptide sequences were only determined for the fraction with the highest potency in term of ACE-inhibitory, hydroxyl radical scavenging, and metal-chelating activities, i.e., HB-IV obtained from ultrafiltration.

### 3.3. Identification of the Peptide Sequences in HB-IV Fraction

A total of 145 peptide sequences in the range of 2 to 9 amino acids with MW from 131 to 921 Da were detected in the HB-IV fraction. The most abundant peptides sequences detected in the HB-IV fraction were of tetrapeptides (54.05%), followed by pentapeptides (18.24%), and hexapeptides (9.46%). After matching to a bioactive peptide database (BIOPEP), only eight sequences (KL, LR, LQ, FL, TL, KY, YVL, and LVLL) matched with sequences demonstrating ACE-inhibitory and antioxidant activities in the database, while the remaining were novel unreported peptides. Interestingly, 105 of the novel peptides have ACE-inhibitory and antioxidant peptides embedded within its sequences, as shown in Supplementary Material (Table S1). For example, AGPL peptide contained sequences of five ACE-inhibitory peptides i.e., GPL, GP, PL, AG, and AGP [36], suggesting the potential of its ACE-inhibitory effect. In addition to the embedded bioactive peptides, some peptides, such as TLPF, LYLF, and LGPL, also have the common feature of antihypertensive peptides, i.e., phenylalanine (F), tyrosine (Y), and/or proline (*p*) located at the C-terminal. This feature has been reported in ACE-inhibitory peptides isolated from cuttlefish (VELYP, IC50: 5.22 µM) [73], snakehead muscle (LYPPP, IC50: 1.3 µM) [41], and salmon by-product (FNVPLYE, IC50: 6.79 mg/mL) [74]. Out of the 105 peptides, 6 peptides (FDLK, FCVH, LPML, LSVLK, DLVDLK, and FELNVT) displayed similarity with antioxidant peptides reported in the BIOPEP database. Their sequence consisted of hydrophobic and aromatic amino acids, which are the common features of antioxidant peptides [21,51,75]. Antioxidant peptides with these features had been isolated from the sea squirt (LEW and YYPYQL) [34] and red stingray (IEPH) [64].

Since it is economically impractical to synthesize all the 105 peptides, PeptideRanker was used for scoring the identified peptides based on the prediction of their bioactivity. Generally, a sequence is considered potentially bioactive when the logarithm assigns a score of at least of 0.5 [76]. Based on the PeptideRanker scores, 42 out of 105 identified peptides have a high potential of being bioactive (score > 0.5) (Table 5). The presence of known bioactive peptide sequences with difference bioactivities (matching BIOPEP database) in HB-IV would have contributed to the collective biological benefits reported in this fraction. In addition to the known peptides, it is predicted that the unknown peptides may also have participated in the bioactivities as they contained potential ACE-inhibitory and antioxidant peptides within their sequences. This however needs to be further confirmed by more studies in the future.

### 3.4. Stability of HB-IV Fraction against In Vitro Stimulated Gastrointestinal Digestion

**Figure 5:**
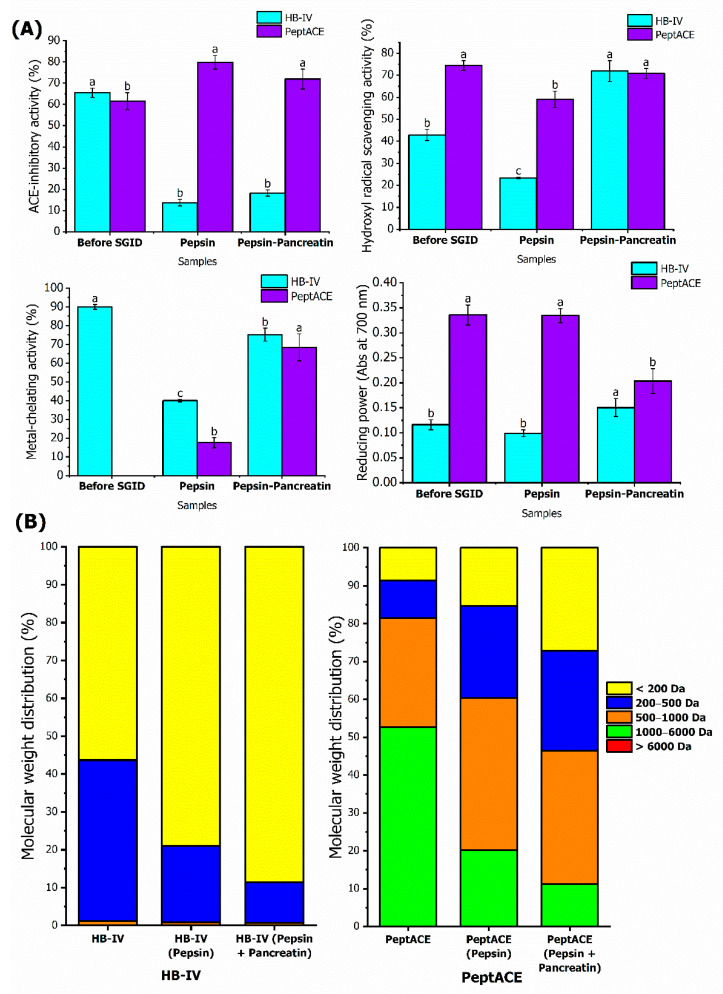
The effect of in vitro stimulated gastrointestinal digestion on HB-IV and PeptACE. (**A**) The bioactivities of HB-IV and PeptACE during SGID. (**B**) The molecular weight distribution of HB-IV and PeptACE before and after SGID. The values represent the mean ± SD. Means without a common letter (a–c) on the bars indicate significant difference at *p* < 0.05.

The ability to survive gastrointestinal digestion is essential for maintenance of the bioactivities of peptides in vivo. Some peptides showing bioactivity in vitro failed to exert effect in vivo due to possible modifications or degradation of the peptides by the digestion of gastrointestinal enzymes [77]. In vitro stimulated gastrointestinal digestion (SGID) investigates the resistance of the peptides towards gastrointestinal enzymes and predicts the bioavailability of the digested peptides. To evaluate the stability against in vitro stimulated gastrointestinal digestion (SGID), HB-IV fraction was subjected to sequential digestion by pepsin and pancreatin. The stability of PeptACE against SGID was also studied for comparison purposes. The bioactivities of HB-IV and PeptACE prior to gastrointestinal digestion, and after subjecting to pepsin and pancreatin digestion are shown in Figure 5A.

As depicted in Figure 5A, ACE-inhibitory activity of HB-IV dropped 4.8 times to 13.70% (*p* < 0.05) after going through pepsin digestion, and after increased slightly (*p* > 0.05) until the end of SGID. This showed that SGID resulted in peptides with weaker ACE-inhibition capacity. PeptACE has a completely opposite trend to HB-IV, its ACE-inhibition potency increased significantly (*p* < 0.05) after pepsin digestion, and maintained after pancreatin digestion. At the end of SGID, HB-IV was reported to have four times lower ACE-inhibition capacity than PeptACE, even though both exhibited similar inhibitory activity prior to SGID. The improvement of ACE-inhibitory activity in PeptACE was expected as more potent di- and tripeptides, i.e., IW (IC50: 0.63 µg/mL) and IKP (IC50: 0.61 µg/mL) were released from their parental peptides, IWHHT (IC50: 3.5 µg/mL) and IKPLNY (IC50: 32 µg/mL), respectively, after digestion by gastrointestinal proteases [78]. Other than ACE-inhibitory activity, the metal-chelating activity of HB-IV was also reduced by 55% because of digestion by pepsin. However, this chelating ability was later recovered by over 80% upon subsequent pancreatin digestion, but the activity was still lower than prior to SGID. Contrary to HB-IV, SGID clearly improved the metal- chelating ability of PeptACE whereby it only started to be observed after pepsin digestion, and the capacity continuously increased until the end of digestion. Both the PeptACE and HB-IV have similar chelating capacity at the end of SGID (*p* > 0.05). It is worth noting that after digestion, the chelating ability of HB-IV at 0.2 mg/mL (75.18%) is comparable to the SGID-digest of carp skin gelatine at 10 mg/mL (73.25%).

During the sequential gastrointestinal digestion, the peptides in HB-IV were further broken down by pepsin and pancreatin resulting in smaller peptides or free amino acids, which could either exhibit stronger or weaker bioactivities. The peptides found in HB-IV were mainly made up of peptides with MW 200–500 Da and below 200 Da, but SGID caused the peptides in 200–500 Da to further breakdown leading to a noticeable increase in peptides with MW below 200 Da (possibly dipeptides or free amino acids) (Figure 5B). This could also mean that the potent ACE-inhibitory peptides were most probably attributed to the bigger peptides with MW ranging from 200–500 Da. This observation is in accordance with Quirós et al. [56], who reported the ACE-inhibitory capacity of VLGPVRGPFP was significantly reduced by four times after it was hydrolysed into two smaller fragments peptides (VLGPV and LGPVR) in the digestive process. The reduction of ACE-inhibitory activity after in vitro gastrointestinal digestion was also reported in fish species, such as tilapia [79] and the giant catfish [80]. Some other studies reported contradictory findings, whereby the ACE-inhibitory potential of hydrolysate was unaffected by the digestion of gastrointestinal enzymes. Examples were hydrolysate derived from grass carp [70] and salmon [23].

Unlike ACE-inhibitory and metal-chelating activities, SGID exerted a positive impact on hydroxyl radical scavenging capacity and the reducing power of HB-IV. The hydroxyl radical scavenging activity of both HB-IV and PeptACE was reduced by 45% and 21%, respectively, after pepsin digestion, but it was raised after SGID. It is worth noting that even though the scavenging activity of HB-IV is 40% lower than PeptACE prior to digestion, further pancreatin digestion manifested the scavenging activity of HB-IV, bringing its capacities up to a level similar to PeptACE. The reducing power of both the HB-IV and PeptACE showed no substantial changes during pepsin digestion (*p* > 0.05), but further pancreatin digestion influenced their reducing power differently. The reducing power of HB-IV was enhanced to a level that was 29.54% higher than before SGID (*p* < 0.05), whereas the reducing power of PeptACE was significantly reduced by 39.38% (*p* < 0.05). Though SGID lowered the reducing power of PeptACE, its reducing potential was still reported to be higher than HB-IV (0.2035 absorbance unit vs. 0.1505 absorbance unit) (*p* < 0.05).

Overall, the changes in peptides profiles by SGID further improved the antioxidant potential (hydroxyl radical scavenging activity and reducing power) of HB-IV. During the earlier stage of SGID, pepsin treatment may disrupt the structure of potent antioxidant peptides in HB-IV and reduce its capabilities to scavenge hydroxyl radicals and chelate ferrous ions. However, these antioxidant capacities were significantly improved by further pancreatin digestion suggesting that pancreatin caused cleavage of the peptides to release lower molecular weight structures with high antioxidant affinity groups, such as hydrophobic or aromatic amino acids with imidazole (histidine) and indole ring (tryptophan) [77,81]. The improvement of antioxidant capacities after SGID was also reported in stripped weakfish [82], blacktip shark [83], and brownstripe red snapper [84]. The SGID process also changed the molecular weight distribution of PeptACE (Figure 4B). The majority of the peptides being cleaved during SGID were those with MW range of 1000–6000 Da, resulting in a higher proportion of peptides with MW below 1000 Da. Since PeptACE is a supplement targeting antihypertensive effect, no data on its antioxidant performance after SGID is published to date. It can only be assumed that in addition to ACE-inhibitory activity, the peptides generated in PeptACE after SGID also contributed to strong hydroxyl radical scavenging and metal-chelating capacities seen in this study. Based on the changes in the MW distributions (Figure 4), a new set of peptides were generated in HB-IV and PeptACE following in vitro SGID. The peptide sequences of these newly formed peptides need to be identified in the future in order to have a better understanding on how the changes of peptide profiles affected the bioactivities throughout the pepsin-pancreatin digestion.

### 3.5. Stability of HB-IV Fraction against ACE

The stability of peptides against ACE was important to ensure that the peptides retained their in vivo antihypertensive effect. Some peptides were reported to exhibit ACE-inhibitory activity in vitro but not in vivo because the peptides were degraded by ACE. According to Fujita et al. [85], ACE-inhibitory peptides are classified into three categories based on the alteration in their ACE-inhibitory activity after hydrolysis by ACE, which include true inhibitor (ACE-inhibitory activity did not show significant changes after pre-incubation with ACE), real substrate (ACE-inhibitory activity decreased after pre-incubation with ACE), and pro-drug type (ACE-inhibitory activity increased after pre-incubation with ACE). A study demonstrated by Fujita et al. [85] also reported that only peptides belong to true inhibitor or pro-drug exhibited antihypertensive effect in spontaneous hypertensive rat (SHR).

As shown in Table 6, the IC50 value of HB-IV increased by five times after pre-incubation with ACE, indicating the reduction of its ACE-inhibitory potency. The reduction of inhibitory activity after pre-incubation with ACE shows that HB-IV acts as substrate to ACE, and the peptides were hydrolysed by ACE resulting in weaker inhibitory activity. However, the inhibitory activity remained unchanged after HB-IV was subjected to SGID. This showed that the new peptides formed after pepsin and pancreatin digestion are now resistant to ACE hydrolysis and capable of exerting an antihypertensive effect. This finding suggests the digestion of HB-IV by gastrointestinal enzymes is important for the onset of its antihypertensive effect because these enzymes could turn the peptides in HB-IV from substrate into a true inhibitor. A study by Fujita et al. [85] also showed the importance of gastrointestinal enzymes to activate the antihypertensive effect of peptide isolated from bonito hydrolysate. The peptide IVGRPRHQG only displayed antihypertensive effect in SHR when it was fed orally instead of by intravenous injection. This was because using intravenous injection, IVGRPRHQG was directly hydrolysed by ACE resulting in peptides which could not exhibit antihypertensive effect. However, upon digestion by pepsin and trypsin, two true inhibitors, HQG and IVGPRP which were resistant to ACE, were produced. Therefore, in order for HB-IV to exhibit blood pressure lowering effect, it must be introduced through oral supplementation instead of intravenous injection.

The ACE-inhibitory activity of PeptACE, both before and after SGID, were also increased after pre-incubation with ACE, categorising them as pro-drug (Table 6). This observation was also reported by several authors who studied PeptACE [38,78,86]. According to Fujita et al. [85] and Yokoyama et al. [78], the increment of ACE-inhibitory potency was because two peptides, i.e., LKPNM (IC50: 2.4 µM) and IWHTT (IC50: 5.8 µM) in PeptACE, were cleaved by ACE into LKP (IC50: 0.32 µM) and IWH (IC50: 3.5 µM), respectively, that contributed to significantly higher inhibitory potency.

### 3.6. ACE-Inhibitory Kinetics of HB-IV Fraction

**Figure 6:**
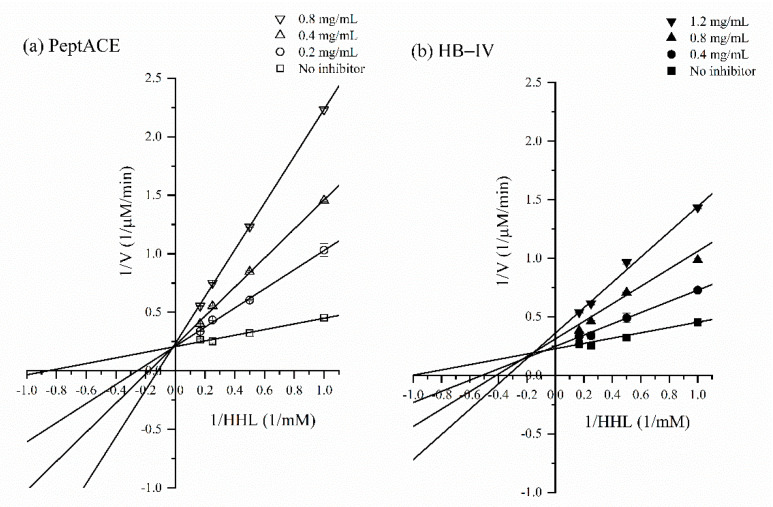
The Lineweaver–Burk plots for the inhibition of ACE by different concentrations of (**a**) PeptACE and (**b**) HB-IV.

The mode of ACE-inhibition of HB-IV was analysed using the maximum enzyme rate (Vmax) and Michaelis-Menten constant (Km) values obtained from Lineweaver-Burk plot, as depicted in Figure 6. It can be noted that HB-IV and PeptACE exhibited different inhibition patterns, though both have similar IC50 values as mentioned earlier in Section 3.2.1 (Table 1). This showed that peptides responsible for ACE-inhibition in HB-IV and PeptACE were most probably of different sequences. All the lines in Figure 6a intersected at different *x*-axis but have the same intersection at *y*-axis. This means that with the presence of PeptACE, the Vmax of ACE was unaffected, whilst the Km values increased as compared to the values obtained from reaction without an inhibitor. This is the characteristic of a competitive inhibitor as increasing the PeptACE concentration had caused more hippuryl-histidyl-leucine (HHL) (higher Km value) is needed to achieve half of the maximum velocity of ACE. The reason being the peptides in PeptACE would compete with HHL for the active site of ACE. This also suggested that the shape of ACE-inhibitor in PeptACE might mimic HHL, and, therefore, is capable of binding to an active site of ACE. The competitive inhibition of PeptACE was also reported in study by Cinq-Mars et al. [38].

On the other hand, HB-IV showed a mixed type of inhibition mode as its presence affected both the Vmax and Km values as shown by the lines with different intersections in y- and *x*-axis (Figure 6b). Mixed mode inhibition happened when the inhibitor demonstrated more than one inhibition modality [87]. The decrease in Vmax and increase in Km values indicate that HB-IV had reduced the maximum velocity of ACE and, at the same time, a higher concentration of HHL is also needed. This suggested that the peptides in HB-IV can inhibit ACE activity in two ways, either by binding to the active site or the non-active site of ACE [39]. When the peptides bind to the non-active site of ACE, the conformation of the ACE will be altered and prevent the HHL from binding to ACE. This process interfered with the turnover rate of an enzyme and thus a lower Vmax value will be observed if more of the inhibitor is present. The pattern of inhibition expressed by HB-IV is similar to the work on salmon by-product [74], catfish [39], and tilapia skin [1].

There was no correlation between the mode of inhibition and the ACE-inhibitory capacity. Some studies have reported peptides with mixed type of inhibition mode have higher ACE-inhibition capacity than peptides with non-competitive or competitive inhibition mode and vice versa. For example, among two peptides isolated from catfish myofibrillar, the peptide GPPP (IC50: 0.86 µM) with mixed type inhibition exhibited 40% higher ACE-inhibitory activity than peptide IEKPP (IC50: 1.2 µM) which inhibits ACE in a competitive manner [39]. In another study, Ono et al. [88] reported that the non-competitive peptide, MW (IC50: 9.8 μM) purified from salmon muscle, exhibited 39-fold higher ACE-inhibitory activity than competitive peptide, LP (IC50: 383.2 μM). Forghani et al. [87] reported that the efficiency of ACE-inhibition was dependent on the sequences of the peptides instead of inhibition mode because even if peptides have the same inhibition mode, their ACE-inhibitory potency would still vary greatly. For example, both the peptides EVSQGRP (IC50: 0.05 μM) and SAAVGSP (IC50: 1.71 μM) also exhibited mixed types of inhibition, but the former was a better ACE-inhibitor than the latter.

## 4. Conclusions

This current research has explored the potential of by-products (fish head and bones) from TGGG in producing ACE-inhibitory and antioxidant peptides. ACE-inhibitory and antioxidant peptides were successfully isolated from the heads and bones hydrolysate of hybrid groupers using ultrafiltration and chromatographic techniques. Fraction of molecular weight below 1 kDa obtained from ultrafiltration showed the strongest ACE-inhibitory, hydroxyl radical scavenging, and metal-chelating activities (*p* < 0.05). It was noticed that purified fractions with high acidic and hydrophobic amino acids content (75–200 Da) exhibited the highest ACE-inhibitory and antioxidant activities (*p* < 0.05). The findings suggested that ultrafiltration alone was sufficient to isolate potent ACE-inhibitory and antioxidant peptides from the hydrolysate as further separation by chromatography caused significant reduction in the potency. The most potent ultrafiltrated fraction (HB-IV) was made up of 145 peptides with MW below 1 kDa, of which 95% of the identified peptides were novel and had not been recorded in the bioactive peptides database, BIOPEP. This fraction exhibited ACE-inhibitory capacity that is comparable to PeptACE (a commercial antihypertensive supplement). In contrast to ACE-inhibitory and metal-chelating activity, the hydroxyl radical scavenging and reducing power of HB-IV were increased following in vitro gastrointestinal digestion, suggesting its better potential as a radical scavenging, and reducing agent. However, further studies to investigate the effectiveness of HB-IV to ameliorate oxidative stress and reducing blood pressure using in vivo model are needed in the future.

## Figures and Tables

**Table 1:** The ACE-inhibitory activity, antioxidant activities, protein content, and yield of ultrafiltration fractions obtained from Al-HB.

Fraction	MW (kDa)	ACE-Inhibitory Activity (IC50, mg/mL)	Hydroxyl Radical Scavenging Activity (EC50, mg/mL)	Reducing Power * (Abs at 700 nm)	Metal-chelating Activity (EC50, ug/mL)	Protein Content (mg/g)	Yield
HB-I	>5	1.19 ± 0.17 ^a^	6.97 ± 0.70 ^a^	0.352 ± 0.039 ^a^	156.23 ± 18.00 ^a^	104.34 ± 2.37 ^a^	25.10 ± 0.78 ^a^
HB-II	3–5	0.39 ± 0.06 ^b^	6.74 ± 0.71 ^a^	0.264 ± 0.010 ^b^	83.24 ± 9.99 ^b^	83.25 ± 2.08 ^b^	19.87 ± 0.21 ^b^
HB-III	1–3	0.44 ± 0.10 ^b^	5.90 ± 0.60 ^a^	0.203 ± 0.024 ^b^	34.34 ± 3.20 ^c^	77.15 ± 4.01 ^b^	19.27 ± 0.32 ^b^
HB-IV	<1	0.28 ± 0.03 ^b^	3.48 ± 0.14 ^b^	0.116 ± 0.007 ^c^	34.07 ± 2.24 ^c^	37.41 ± 0.30 ^c^	14.37 ± 0.57 ^c^
PeptACE ^^^		0.22 ± 0.03 ^b^	0.44 ± 0.02 ^c^	0.336 ± 0.024 ^a^	>1000	ND	ND
Ascorbic acid		ND	1.57 ± 0.20 ^c^	0.928 ± 0.027 ^#^	ND	ND	ND
EDTA		ND	ND	ND	4.34 ± 0.43 ^d^	ND	ND

Values are mean ± SD, *n* = 3. Means without a common superscript letter (a–d) within the same column indicate significant difference at *p* < 0.05. * Sample concentration used for analysis was 1 mg/mL. ^^^ An antihypertensive supplement composed of fish peptides manufactured by Natural Factors Nutritional Product Ltd. (Coquitlam, Canada). ^#^ Ascorbic acid concentration used for reducing power determination was 0.005 mg/mL. ND: not determined.

**Table 2:** Amino acid composition (expressed as % of total amino acids) of Al-HB and its peptide fractions.

Amino Acids	Amino Acid Composition (% Total Amino Acids)					
	Al-HB	HB-I	HB-II	HB-III	HB-IV	PeptACE
Asp	8.00 ± 0.15 ^d^	7.87 ± 0.29 ^d^	9.49 ± 0.12 ^b^	8.75 ± 0.15 ^c^	7.79 ± 0.30 ^d^	10.75 ± 0.06 ^a^
Glu	13.89 ± 0.27 ^cd^	13.08 ± 0.48 ^d^	15.10 ± 0.96 ^bc^	15.93 ± 0.36 ^ab^	15.55 ± 0.41 ^ab^	16.82 ± 0.12 ^a^
Ser	4.04 ± 0.13 ^c^	3.86 ± 0.19 ^c^	3.82 ± 0.28 ^c^	4.64 ± 0.17 ^b^	5.31 ± 0.25 ^a^	4.19 ± 0.02 ^bc^
His	1.98 ± 0.24 ^cd^	1.68 ± 0.23 ^d^	2.26 ± 0.17 ^bcd^	2.61 ± 0.43 ^bc^	2.90 ± 0.33 ^b^	4.97 ± 0.20 ^a^
Gly	11.83 ± 0.38 ^b^	15.46 ± 0.80 ^a^	7.78 ± 0.16 ^c^	6.20 ± 0.24 ^d^	8.12 ± 1.03 ^c^	4.37 ± 0.04 ^e^
Thr	4.04 ± 0.11 ^cd^	3.48 ± 0.24 ^d^	4.20 ± 0.25 ^bc^	4.94 ± 0.14 ^a^	4.51 ± 0.41 ^abc^	4.72 ± 0.07 ^ab^
Arg	7.88 ± 0.19 ^ab^	8.81 ± 0.33 ^a^	7.76 ± 0.86 ^ab^	7.13 ± 0.21 ^bc^	5.66 ± 0.21 ^d^	6.15 ± 0.09 ^cd^
Ala	8.17 ± 0.15 ^b^	8.09 ± 0.28 ^b^	6.83 ± 0.26 ^c^	8.17 ± 0.18 ^b^	13.41 ± 0.78 ^a^	6.84 ± 0.05 ^c^
Tyr	2.46 ± 0.06 ^c^	1.68 ± 0.04 ^d^	2.74 ± 0.15 ^b^	3.78 ± 0.04 ^a^	3.57 ± 0.11 ^a^	2.72 ± 0.04 ^b^
Val	4.54 ± 0.10 b^c^	3.97 ± 0.10 ^c^	5.16 ± 0.37 ^ab^	5.78 ± 0.12 ^a^	4.48 ± 0.50 ^bc^	4.63 ± 0.09 ^bc^
Met	2.47 ± 0.32 ^c^	1.98 ± 0.10 ^d^	2.41 ± 0.01 ^c^	3.44 ± 0.05 ^a^	3.80 ± 0.14 ^a^	2.96 ± 0.09 ^b^
Trp	0.73 ± 0.15 ^b^	0.93 ± 0.12 ^b^	1.83 ± 0.12 ^a^	0.95 ± 0.27 ^b^	1.01 ± 0.17 ^b^	0.71 ± 0.02 ^b^
Phe	4.07 ± 0.05 ^bc^	3.64 ± 0.19 ^c^	4.29 ± 0.12 ^abc^	5.04 ± 0.07 ^a^	4.66 ± 0.63 ^ab^	3.76 ± 0.06 ^c^
Ile	3.90 ± 0.12 ^c^	3.14 ± 0.09 ^d^	4.82 ± 0.12 ^b^	5.27 ± 0.06 ^a^	3.68 ± 0.30 ^c^	4.58 ± 0.04 ^b^
Leu	6.88 ± 0.12 ^c^	5.36 ± 0.16 ^d^	7.91 ± 0.24 ^b^	9.44 ± 0.15 ^a^	9.42 ± 0.09 ^a^	8.14 ± 0.05 ^b^
Lys	6.31 ± 1.02 ^bc^	3.52 ± 0.78 ^d^	7.37 ± 0.90 ^ab^	7.13 ± 1.27 ^bc^	4.66 ± 0.81 ^cd^	10.04 ± 1.03 ^a^
Hyp	2.36 ± 0.13 ^b^	3.39 ± 0.42 ^a^	1.36 ± 0.14 ^c^	0.84 ± 0.16 ^c^	1.41 ± 0.28 ^c^	ND
Pro	6.47 ± 0.31 ^b^	10.07 ± 1.98 ^a^	4.84 ± 0.40 ^bc^	2.90 ± 0.30 ^cd^	1.53 ± 0.14 ^d^	3.63 ± 0.39 ^cd^
HAA	33.21 ± 0.46 ^d^	28.79 ± 0.65 ^e^	36.01 ± 0.44 ^c^	41.85 ± 0.48 ^b^	44.28 ± 1.04 ^a^	34.34 ± 0.21 ^d^
PCAA	16.18 ± 1.08 ^bcd^	14.00 ± 0.50 ^cd^	17.39 ± 1.49 ^b^	16.86 ± 1.29 ^bc^	13.29 ± 1.02 ^d^	21.17 ± 0.74 ^a^
NCAA	21.89 ± 0.42 ^cd^	20.94 ± 0.77 ^d^	24.59 ± 0.85 ^b^	24.68 ± 0.50 ^b^	23.46 ± 0.49 ^bc^	27.57 ± 0.18 ^a^
AroAA	7.25 ± 0.09 ^c^	6.25 ± 0.13 ^d^	8.87 ± 0.06 ^b^	9.76 ± 0.23 ^a^	9.28 ± 0.48 ^ab^	7.18 ± 0.06 ^c^
BCAA	15.31 ± 0.32 ^c^	12.47 ± 0.33 ^d^	17.89 ± 0.36 ^b^	20.49 ± 0.31 ^a^	17.68 ± 0.46 ^b^	17.36 ± 0.11 ^b^

Values are mean ± SD, *n* = 3. Means without a common superscript letter (a–e) across the same row indicate significant difference at *p* < 0.05. Amino acids are described by the three-letter abbreviation. Al-HB: Alcalase-treated bone hydrolysate; HB-I (MW > 5 kDa); HB-II (MW 3–5 kDa); HB-III (MW 1–3 kDa); HB-IV (MW < 1 kDa); ND: not detected; hydrophobic amino acids (HAA) = alanine, tyrosine, valine, methionine, tryptophan, phenylalanine, isoleucine, leucine; positively charged amino acids (PCAA) = histidine, arginine, lysine; negatively charged amino acids (NCAA) = aspartic acid, glutamic acid; aromatic amino acids (AroAA) = tyrosine, tryptophan, phenylalanine; branched amino acids (BCAA) = leucine, valine, isoleucine.

**Table 3:** The amino acids composition (%) of the anion exchange peptide fractions.

Amino Acids	Amino Acid Composition (% Total Amino Acids)		
	F1	F2	F3
Asp	2.49 ± 0.20 ^c^	9.10 ± 0.29 ^a^	7.89 ± 0.45 ^b^
Glu	7.33 ± 0.63 ^c^	27.19 ± 1.14 ^a^	29.30 ± 1.50 ^a^
Ser	5.85 ± 0.42 ^b^	7.92 ± 0.34 ^a^	4.68 ± 0.32 ^c^
His	5.90 ± 0.57 ^a^	ND	ND
Gly	17.61 ± 0.39 ^a^	12.22 ± 0.83 ^b^	13.02 ± 2.30 ^b^
Thr	2.63 ± 0.27 ^a^	1.54 ± 0.23 ^b^	0.91 ± 0.16 ^c^
Arg	13.25 ± 1.20 ^ab^	10.29 ± 1.15 ^b^	15.33 ± 1.74 ^a^
Ala	25.60 ± 3.13 ^a^	2.56 ± 0.35 ^b^	1.29 ± 0.18 ^b^
Tyr	3.68 ± 0.55 ^c^	6.70 ± 0.70 ^b^	8.24 ± 0.44 ^a^
Val	2.31 ± 0.42 ^a^	2.17 ± 0.31 ^a^	1.33 ± 0.24 ^b^
Met	1.39 ± 0.26 ^a^	0.94 ± 0.21 ^a^	0.42 ± 0.04 ^b^
Phe	2.27 ± 0.26 ^a^	2.71 ± 0.50 ^a^	2.75 ± 0.21 ^a^
Ile	1.28 ± 0.07 ^b^	1.82 ± 0.20 ^a^	0.98 ± 0.19 ^b^
Leu	6.21 ± 0.61 ^ab^	7.36 ± 0.34 ^a^	4.77 ± 0.79 ^b^
Lys	2.21 ± 0.10 ^c^	7.47 ± 0.04 ^b^	9.08 ± 1.08 ^a^
HAA	42.74 ± 2.44 ^a^	24.27 ± 1.31 ^b^	19.78 ± 1.16 ^c^
PCAA	21.36 ± 1.37 ^b^	17.75 ± 1.16 ^c^	24.41 ± 0.92 ^a^
NCAA	9.82 ± 0.83 ^b^	36.29 ± 1.69 ^a^	37.19 ± 1.92 ^a^
AroAA	5.95 ± 0.35 ^b^	9.42 ± 1.08 ^a^	10.99 ± 0.57 ^a^
BCAA	8.88 ± 0.82 ^a^	10.12 ± 0.45 ^a^	6.16 ± 0.93 ^b^

Values are mean ± SD, *n* = 3. Means without a common superscript letter (a–c) across the same row indicate significant difference at *p* < 0.05. ND: not detected; hydrophobic amino acids (HAA) = alanine, tyrosine, valine, methionine, tryptophan, phenylalanine, isoleucine, leucine; positively charged amino acids (PCAA) = histidine, arginine, lysine; negatively charged amino acids (NCAA) = aspartic acid, glutamic acid; aromatic amino acids (AroAA) = tyrosine, tryptophan, phenylalanine; branched amino acids (BCAA) = leucine, valine, isoleucine.

**Table 4:** Summary for bioactivities of the potent fraction collected after a series of purification steps on bone hydrolysate.

Fractions	Step	Bioactivities			
		ACE-Inhibitory Activity (IC50, mg/mL)	Hydroxyl Radical Scavenging Activity (EC50, mg/mL)	Reducing Power * (Abs at 700 nm)	Metal-chelating Activity (EC50, mg/mL)
Al-HB		0.84 ± 0.17	6.82 ± 0.63	0.239 ± 0.026	0.042 ± 0.005
HB-IV	Ultrafiltration	0.28 ± 0.03	3.48 ± 0.14	0.116 ± 0.007	0.034 ± 0.002
F1	Anion exchange chromatography	3.18 ± 0.53	4.45 ± 0.72	0.136 ± 0.008	5.15 ± 0.98
F1-B	Gel filtration chromatography	5.76 ± 0.78	9.01 ± 1.98	0.039 ± 0.004	5.89 ± 0.60

Values are mean ± SD, *n* = 3. * Sample concentration used for analysis was 1 mg/mL.

**Table 5:** Potential bioactive peptides in HB-IV fraction as ranked using PeptideRanker.

Identified Peptide Sequences *	Observed Mass (Da)	Identified Peptide Sequences *	Observed Mass (Da)
RL	287.19	FDLK	521.28
LL	244.18	FCVH	504.22
ALLL	428.29	LPML	472.27
LRL	400.28	LKLF	519.34
LGGL	358.22	MLCM	496.18
AGPL	356.21	LGFL	448.27
PCLN	445.20	MMVF	542.22
LLGL	414.28	MPFE	522.21
LAFL	463.28	LGSF	422.22
LSFL	478.28	FAGL	406.22
LGLL	414.28	MLLP	472.27
FLGM	466.23	MPDLR	630.32
TLPF	476.26	FADTF	599.26
LPAL	412.27	MGGVF	509.23
AFLK	477.30	MPLLM	619.31
DPLL	456.26	LDAGF	521.25
LGPL	398.25	GPAGPL	510.28
LNFL	505.29	YPGLWMR	921.45
VALF	448.27	YPGLADMR	921.44
ALFL	462.28	PPACLTAL	784.42
LYLF	554.31	LLMLLL	714.47

* Amino acids are designated using their one-letter codes.

**Table 6:** ACE-inhibitory activity of HB-IV and PeptACE with and without pre-incubation with ACE.

Samples	ACE-Inhibitory Activity (IC50, mg/mL)		Inhibitor Category
	Without Pre-Incubation	With Pre-Incubation	
HB-IV	0.28 ± 0.03 ^b^	1.52 ± 0.11 ^a^	Substrate
HB-IV (SGID)	1.76 ± 0.28 ^a^	1.96 ± 0.22 ^a^	True inhibitor
PeptACE	0.22 ± 0.03 ^a^	0.15 ± 0.01 ^b^	Pro-drug
PeptACE (SGID)	0.19 ± 0.04 ^a^	0.13 ± 0.02 ^b^	Pro-drug

Values are mean ± SD, *n* = 3. Means without a common superscript letter (a, b) across the same row indicate significant difference at *p* < 0.05 using independent sample t-test. HB-IV: ultrafiltrated fraction with MW below 1 kDa; HB-IV (SGID): ultrafiltrated fraction with MW below 1 kDa after in vitro gastrointestinal digestion.

## Data Availability

The authors confirm that the data supporting the findings of this study are available within the article and Supplementary Materials.

## Supplementary Materials

The following supporting information can be downloaded at: https://www.mdpi.com/article/10.3390/foods11243991/s1, Table S1: List of peptide sequences with potential ACE-inhibitory and antioxidant activities identified in HB-IV fraction.

## References

[1] Chen J., Ryu B., Zhang Y., Liang P., Li C., Zhou C., Yang P., Hong P., Qian Z. (2020). Comparison of an Angiotensin-I-Converting Enzyme Inhibitory Peptide from Tilapia (*Oreachromix niloticus*) with Captopril: Inhibition Kinetics, in Vivo Effect, Stimulated Gastrointestinal Digestion and a Molecular Docking Study. J. Sci. Food Agric..

[2] Ishak N.H., Shaik M.I., Yellapu N.K., Howell N.K., Sarbon N.M. (2021). Purification, Characterization and Molecular Docking Study of Angiotensin-I Converting Enzyme (ACE) Inhibitory Peptide from Shortfin Scad (*Decapterus macrosoma*) Protein Hydrolysate. J. Food Sci. Technol..

[3] Bashir K.M.I., Sohn J.H., Kim J.-S., Choi J.-S. (2020). Identification and Characterization of Novel Antioxidant Peptides from Mackerel (*Scomber japonicus*) Muscle Protein Hydrolysates. Food Chem..

[4] Najafian L., Babji A.S. (2014). Production of Bioactive Peptides Using Enzymatic Hydrolysis and Identification Antioxidative Peptides from Patin (*Pangasius sutchi*) Sarcoplasmic Protein Hydolysate. J. Funct. Foods.

[5] Rivero-Pino F., Espejo-Carpio F.J., Guadix E.M. (2020). Production and Identification of Dipeptidyl Peptidase IV (DPP-IV) Inhibitory Peptides from Discarded *Sardine pilchardus* Protein. Food Chem..

[6] Dahiya R., Gautam H., Jadon G., Dahiya S., Onuh J.O., Selvamuthukumaran M., Pathak Y.V. (2021). Bioactive Oxazole-Based Cyclopolypeptides from Marine Resources and Their Health Potential. Bioactive Peptides: Production, Bioavailability, Health Potential, and Regulatory Issues.

[7] Charoensiddhi S., Conlon M.A., Franco C.M.M., Zhang W. (2017). The Development of Seaweed-Derived Bioactive Compounds for Use as Prebiotics and Nutraceuticals Using Enzyme Technologies. Trends Food Sci. Technol..

[8] Chalamaiah M., Dinesh Kumar B., Hemalatha R., Jyothirmayi T. (2012). Fish Protein Hydrolysates: Proximate Composition, Amino Acid Composition, Antioxidant Activities and Applications: A Review. Food Chem..

[9] Kumar J. (2013). Epidemiology of Hypertension. Clin. Queries Nephrol..

[10] Kearney P.M., Whelton M., Reynolds K., Muntner P., Whelton P.K., He J. (2005). Global Burden of Hypertension: Analysis of Worldwide Data. Lancet.

[11] World Health Organization (WHO) (2013). A Global Brief on Hypertension.

[12] Ministry of Health (MOH) (2019). National Health and Morbility Survey 2019.

[13] Wijesekara I., Kim S.-K. (2010). Angiotensin-I-Converting Enzyme (ACE) Inhibitors from Marine Resources: Prospects in the Pharmaceutical Industry. Mar. Drugs.

[14] Havelka J., Boerlin H.J., Studer A., Greminger P., Tenschert W., Luescher T., Siegenthaler W., Vetter W., Walger P., Vetter H. (1982). Long-Term Experience with Captopril in Severe Hypertension. Br. J. Clin. Pharmacol..

[15] Kawasaki T., Seki E., Osajima K., Yoshida M., Asada K., Matsui T., Osajima Y. (2000). Antihypertensive Effect of Valyl-Tyrosine, a Short Chain Peptide Derived from Sardine Muscle Hydrolyzate, on Mild Hypertensive Subjects. J. Hum. Hypertens..

[16] Tkaczewska J., Borczak B., Piątkowska E., Kapusta-Duch J., Morawska M., Czech T. (2019). Effect of Protein Hydrolysates from Carp (*Cyprinus carpio*) Skin Gelatine on Oxidative Stress Biomarkers and Other Blood Parameters in Healthy Rats. J. Funct. Foods.

[17] Ch’ng C.L., Senoo S. (2008). Egg and Larval Development of a New Hybrid Grouper, Tiger Grouper *Epinephelus fuscoguttatus* x Giant Grouper, *E. lanceolatus*. Aquac. Sci..

[18] Ching F.F., Othman N., Anuar A., Shapawi R., Senoo S. (2018). Natural Spawning, Embryonic and Larval Development of F2 Hybrid Grouper, Tiger Grouper *Epinephelus fuscoguttatus* × Giant Grouper, *E. lanceolatus*. Int. Aquat. Res..

[19] Department of Fisheries Malaysia Annual Fisheries Statistics 2017. https://www.dof.gov.my/en/resources/fisheries-statistics-i/.

[20] (2018). Department of Fisheries Malaysia. Annual Fisheries Statistics. https://www.dof.gov.my/en/resources/fisheries-statistics-i/.

[21] Yang X.R., Zhao Y.Q., Qiu Y.T., Chi C.F., Wang B. (2019). Preparation and Characterization of Gelatin and Antioxidant Peptides from Gelatin Hydrolysate of Skipjack Tuna (*Katsuwonus pelamis*) Bone Stimulated by in Vitro Gastrointestinal Digestion. Mar. Drugs.

[22] Yi J., De Gobba C., Skibsted L.H., Otte J. (2017). Angiotensin-I Converting Enzyme Inhibitory and Antioxidant Activity of Bioactive Peptides Produced by Enzymatic Hydrolysis of Skin from Grass Carp (*Ctenopharyngodon idella*). Int. J. Food Prop..

[23] Neves A.C., Harnedy P.A., O’Keeffe M.B., Alashi M.A., Aluko R.E., FitzGerald R.J. (2017). Peptide Identification in a Salmon Gelatin Hydrolysate with Antihypertensive, Dipeptidyl Peptidase IV Inhibitory and Antioxidant Activities. Food Res. Int..

[24] Gómez L.J., Gómez N.A., Zapata J.E., López-García G., Cilla A., Alegría A. (2019). In-Vitro Antioxidant Capacity and Cytoprotective/Cytotoxic Effects upon Caco-2 Cells of Red Tilapia (*Oreochromis spp.*) Viscera Hydrolysates. Food Res. Int..

[25] Xu J., Li Y., Regenstein J., Su X. (2017). In Vitro and in Vivo Anti-Oxidation and Anti-Fatigue Effect of Monkfish Liver Hydrolysate. Food Biosci..

[26] Chan P.-T., Matanjun P., Budiman C., Shapawi R., Lee J.-S. (2020). ACE-Inhibitory and Antioxidant Activities of Hydrolysates from the By-Products of Hybrid Grouper (*Epinephelus lanceolatus* × *Epinephelus fuscoguttatus*). Sains Malays..

[27] Girgih A.T., Udenigwe C.C., Li H., Adebiyi A.P., Aluko R.E. (2012). Kinetics of Enzyme Inhibition and Antihypertensive Effects of Hemp Seed (*Cannabis sativa* L.) Protein Hydrolysates. J. Am. Oil Chem. Soc..

[28] Saidi S., Deratani A., Belleville M.P., Amar R. (2014). Ben Antioxidant Properties of Peptide Fractions from Tuna Dark Muscle Protein By-Product Hydrolysate Produced by Membrane Fractionation Process. Food Res. Int..

[29] Raghavan S., Kristinsson H.G. (2009). ACE-Inhibitory Activity of Tilapia Protein Hydrolysates. Food Chem..

[30] Toopcham T., Roytrakul S., Yongsawatdigul J. (2015). Characterization and Identification of Angiotensin I-Converting Enzyme (ACE) Inhibitory Peptides Derived from Tilapia Using *Virgibacillus Halodenitrificans* SK1-3-7 Proteinases. J. Funct. Foods.

[31] Rutherfurd S.M., Gilani G.S. (2009). Amino Acid Analysis. Current Protocols in Protein Science.

[32] Cushman D.W., Cheung H.S. (1971). Spectrophotometric Assay and Properties Of The Angiotensin Converting Enzyme Of The Rabbit Lung. Biochem. Pharmacol..

[33] Lee J.K., Jeon J.K., Byun H.G. (2011). Effect of Angiotensin I Converting Enzyme Inhibitory Peptide Purified from Skate Skin Hydrolysate. Food Chem..

[34] Je J.Y., Lee K.H., Lee M.H., Ahn C.B. (2009). Antioxidant and Antihypertensive Protein Hydrolysates Produced from Tuna Liver by Enzymatic Hydrolysis. Food Res. Int..

[35] Kim S.S., Ahn C.B., Moon S.W., Je J.Y. (2018). Purification and Antioxidant Activities of Peptides from Sea Squirt (*Halocynthia roretzi*) Protein Hydrolysates Using Pepsin Hydrolysis. Food Biosci..

[36] Minkiewicz P., Iwaniak A., Darewicz M. (2019). BIOPEP-UWM Database of Bioactive Peptides: Current Opportunities. Int. J. Mol. Sci..

[37] Khositanon P., Panya N., Roytrakul S., Krobthong S., Chanroj S., Choksawangkarn W. (2021). Effects of Fermentation Periods on Antioxidant and Angiotensin I-Converting Enzyme Inhibitory Activities of Peptides from Fish Sauce by-Products. LWT Food Sci. Technol..

[38] Cinq-Mars C.D., Hu C., Kitts D.D., Li-Chan E.C.Y. (2008). Investigations into Inhibitor Type and Mode, Simulated Gastrointestinal Digestion, and Cell Transport of the Angiotensin I-Converting Enzyme-Inhibitory Peptides in Pacific Hake (*Merluccius productus*) Fillet Hydrolysate. J. Agric. Food Chem..

[39] Ghassem M., Arihara K., Babji A.S. (2012). Isolation, Purification and Characterisation of Angiotensin I-Converting Enzyme-Inhibitory Peptides Derived from Catfish (*Clarias batrachus*) Muscle Protein Thermolysin Hydrolysates. Int. J. Food Sci. Technol..

[40] Lee J.K., Jeon J.-K., Byun H.-G. (2014). Antihypertensive Effect of Novel Angiotensin I Converting Enzyme Inhibitory Peptide from Chum Salmon (*Oncorhynchus keta*) Skin in Spontaneously Hypertensive Rats. J. Funct. Foods.

[41] Ghassem M., Babji A.S., Said M., Mahmoodani F., Arihara K. (2014). Angiotensin I-Converting Enzyme Inhibitory Peptides from Snakehead Fish Sarcoplasmic Protein Hydrolysate. J. Food Biochem..

[42] Zhou D.-Y., Tang Y., Zhu B.-W., Qin L., Li D.-M., Yang J.-F., Lei K. (2012). Antioxidant Activity of Hydrolysates Obtained from Scallop (*Patinopecten yessoensis*) and Abalone (*Haliotis discus hannai* Ino) Muscle. Food Chem..

[43] Chi C.F., Wang B., Hu F.Y., Wang Y.M., Zhang B., Deng S.G., Wu C.W. (2014). Purification and Identification of Three Novel Antioxidant Peptides from Protein Hydrolysate of Bluefin Leatherjacket (*Navodon septentrionalis*) Skin. Food Res. Int..

[44] Je J.-Y., Park P.-J., Kwon J.Y., Kim S.-K. (2004). A Novel Angiotensin I Converting Enzyme Inhibitory Peptide from Alaska Pollack (*Theragra chalcogramma*) Frame Protein Hydrolysate. J. Agric. Food Chem..

[45] Onuh J.O., Girgih A.T., Malomo S.A., Aluko R.E., Aliani M. (2015). Kinetics of in Vitro Renin and Angiotensin Converting Enzyme Inhibition by Chicken Skin Protein Hydrolysates and Their Blood Pressure Lowering Effects in Spontaneously Hypertensive Rats. J. Funct. Foods.

[46] Norris R., FitzGerald R.J., Hernandez-Ledesma B., Hsieh C.-C. (2013). Antihypertensive Peptides from Food Proteins. Bioactive Food Peptides in Health and Disease.

[47] Zhang P., Chang C., Liu H., Li B., Yan Q., Jiang Z. (2020). Identification of Novel Angiotensin I-Converting Enzyme (ACE) Inhibitory Peptides from Wheat Gluten Hydrolysate by the Protease of *Pseudomonas aeruginosa*. J. Funct. Foods.

[48] Chi C.F., Wang B., Deng Y.Y., Wang Y.M., Deng S.G., Ma J.Y. (2014). Isolation and Characterization of Three Antioxidant Pentapeptides from Protein Hydrolysate of Monkfish (*Lophius litulon*) Muscle. Food Res. Int..

[49] Kimatu B.M., Zhao L., Biao Y., Ma G., Yang W., Pei F., Hu Q. (2017). Antioxidant Potential of Edible Mushroom (*Agaricus bisporus*) Protein Hydrolysates and Their Ultrafiltration Fractions. Food Chem..

[50] Pownall T.L., Udenigwe C.C., Aluko R.E. (2010). Amino Acid Composition and Antioxidant Properties of Pea Seed (*Pisum sativum* L.) Enzymatic Protein Hydrolysate Fractions. J. Agric. Food Chem..

[51] Udenigwe C.C., Aluko R.E. (2011). Chemometric Analysis of the Amino Acid Requirements of Antioxidant Food Protein Hydrolysates. Int. J. Mol. Sci..

[52] Girgih A.T., Udenigwe C.C., Aluko R.E. (2011). In Vitro Antioxidant Properties of Hemp Seed (*Cannabis sativa* L.) Protein Hydrolysate Fractions. J. Am. Oil Chem. Soc..

[53] Je J.Y., Kim S.Y., Kim S.K. (2005). Preparation and Antioxidative Activity of Hoki Frame Protein Hydrolysate Using Ultrafiltration Membranes. Eur. Food Res. Technol..

[54] Gianfranceschi G.L., Gianfranceschi G., Quassinti L., Bramucci M. (2018). Biochemical Requirements of Bioactive Peptides for Nutraceutical Efficacy. J. Funct. Foods.

[55] Meisel H., Walsh D.J., Murray B., FitzGerald R.J., Mine Y., Shahidi F. (2006). ACE-Inhibitory Peptides. Nutraceutical Proteins and Peptides in Health and Disease.

[56] Quirós A., del Contreras M.M., Ramos M., Amigo L., Recio I. (2009). Stability to Gastrointestinal Enzymes and Structure-Activity Relationship of β-Casein-Peptides with Antihypertensive Properties. Peptides.

[57] Daskaya-Dikmen C., Yucetepe A., Karbancioglu-Guler F., Daskaya H., Ozcelik B. (2017). Angiotensin-I-Converting Enzyme (ACE)-Inhibitory Peptides from Plants. Nutrients.

[58] Byun H., Kim S. (2002). Structure and Activity of Angiotensin I Converting Enzyme Inhibitory Peptides Derived from Alaskan Pollack Skin. Biochem. Mol. Biol..

[59] Wu J., Aluko R.E., Nakai S. (2006). Structural Requirements of Angiotensin I-Converting Enzyme Inhibitory Peptides: Quantitative Structure-Activity Relationship Study of Di- and Tripeptides. J. Agric. Food Chem..

[60] Nwachukwu I.D., Aluko R.E. (2019). Structural and Functional Properties of Food Protein-Derived Antioxidant Peptides. J. Food Biochem..

[61] Sarmadi B.H., Ismail A. (2010). Antioxidative Peptides from Food Proteins: A Review. Peptides.

[62] Lin H.-M., Deng S.-G., Huang S.-B. (2014). Antioxidant Activities of Ferrous-Chelating Peptides Isolated from Five Types of Low-Value Fish Protein Hydrolysates. J. Food Biochem..

[63] Qian Z.J., Je J.Y., Kim S.K. (2007). Antihypertensive Effect of Angiotensin I Converting Enzyme-Inhibitory Peptide from Hydrolysates of Bigeye Tuna Dark Muscle, *Thunnus Obesus*. J. Agric. Food Chem..

[64] Pan X., Wang Y., Li L., Chi C. (2019). Four Antioxidant Peptides from Protein Hydrolysate of Red Stingray (Dasyatis Akajei) Cartilages: Isolation, Identification, and In Vitro Activity Evaluation. Mar. Drugs.

[65] Moreno-Montoro M., Olalla-herrera M., Rufian-Henares J.Á., Martínez R.G., Miralles B., Bergillos T., Navarro-Alarcón M., Jauregi P. (2017). Antioxidant, ACE-Inhibitory and Antimicrobial Activity of Fermented Goat Milk: Activity and Physicochemical Property Relationship of the Peptide Components. Food Funct..

[66] Ao J., Li B. (2013). Stability and Antioxidative Activities of Casein Peptide Fractions during Simulated Gastrointestinal Digestion *in Vitro*: Charge Properties of Peptides Affect Digestive Stability. Food Res. Int..

[67] Drauz K., Grayson I., Kleemann A., Krimmer H.-P., Leuchtenberger W., Weckbecker C. (2012). Amino Acids. Ullmann’s Encyclopedia of Industrial Chemistry.

[68] Zhu L., Jie C., Tang X., Xiong Y.L. (2008). Reducing, Radical Scavenging, and Chelation Properties of in Vitro Digests of Alcalase-Treated Zein Hydrolysate. J. Agric. Food Chem..

[69] Weng W., Tang L., Wang B., Chen J., Su W., Osako K., Tanaka M. (2014). Antioxidant Properties of Fractions Isolated from Blue Shark (*Prionace glauca*) Skin Gelatin Hydrolysates. J. Funct. Foods.

[70] Chen J., Wang Y., Zhong Q., Wu Y., Xia W. (2012). Peptides Purification and Characterization of a Novel Angiotensin-I Converting Enzyme (ACE) Inhibitory Peptide Derived from Enzymatic Hydrolysate of Grass Carp Protein. Peptides.

[71] Wu H., He H.L., Chen X.L., Sun C.Y., Zhang Y.Z., Zhou B.C. (2008). Purification and Identification of Novel Angiotensin-I-Converting Enzyme Inhibitory Peptides from Shark Meat Hydrolysate. Process Biochem..

[72] Tuz M.A.O., Campos M.R.S. (2017). Purification of *Mucuna pruriens (L)* Peptide Fractions and Evaluation of Their ACE Inhibitory Effect. Biocatal. Agric. Biotechnol..

[73] Balti R., Bougatef A., Sila A., Guillochon D., Dhulster P., Nedjar-arroume N. (2015). Nine Novel Angiotensin I-Converting Enzyme (ACE) Inhibitory Peptides from Cuttlefish (*Sepia officinalis*) Muscle Protein Hydrolysates and Antihypertensive Effect of the Potent Active Peptide in Spontaneously Hypertensive Rats. Food Chem..

[74] Ahn C., Jeon Y., Kim Y., Je J. (2012). Angiotensin I Converting Enzyme (ACE) Inhibitory Peptides from Salmon Byproduct Protein Hydrolysate by Alcalase Hydrolysis. Process Biochem..

[75] Chai T.-T., Law Y.-C., Wong F.-C., Kim S.-K. (2017). Enzyme-Assisted Discovery of Antioxidant Peptides from Edible Marine Invertebrates: A Review. Mar. Drugs.

[76] Zhang P., Roytrakul S., Sutheerawattananonda M. (2017). Production and Purification of Glucosamine and Angiotensin-I Converting Enzyme (ACE) Inhibitory Peptides from Mushroom Hydrolysates. J. Funct. Foods.

[77] Gallego M., Mauri L., Aristoy M.C., Toldrá F., Mora L. (2020). Antioxidant Peptides Profile in Dry-Cured Ham as Affected by Gastrointestinal Digestion. J. Funct. Foods.

[78] Yokoyama K., Chiba H., Yoshikawa M. (1992). Peptide Inhibitors for Angiotensin I-Converting Enzyme from Thermolysin Digest of Dried Bonitot. Biosci. Biotechnol. Biochem..

[79] Thuanthong M., De Gobba C., Sirinupong N., Youravong W., Otte J. (2017). Purification and Characterization of Angiotensin-Converting Enzyme- Inhibitory Peptides from Nile Tilapia (*Oreochromis niloticus*) Skin Gelatine Produced by an Enzymatic Membrane Reactor. J. Funct. Foods.

[80] Ketnawa S., Benjakul S., Martínez-Alvarez O., Rawdkuen S. (2017). Fish Skin Gelatin Hydrolysates Produced by Visceral Peptidase and Bovine Trypsin: Bioactivity and Stability. Food Chem..

[81] Zou T., He T., Li H., Tang H., Xia E. (2016). The Structure-Activity Relationship of the Antioxidant Peptides from Natural Proteins. Molecules.

[82] Lima K.O., da Costa de Quadros C., da Rocha M., de Lacerda J.T.J.G., Juliano M.A., Dias M., Mendes M.A., Prentice C. (2019). Bioactivity and Bioaccessibility of Protein Hydrolyzates from Industrial Byproducts of Stripped Weakfish (Cynoscion Guatucupa). LWT Food Sci. Technol..

[83] Kittiphattanabawon P., Benjakul S., Visessanguan W., Shahidi F. (2012). Gelatin Hydrolysate from Blacktip Shark Skin Prepared Using Papaya Latex Enzyme: Antioxidant Activity and Its Potential in Model Systems. Food Chem..

[84] Khantaphant S., Benjakul S., Kishimura H. (2011). Antioxidative and ACE Inhibitory Activities of Protein Hydrolysates from the Muscle of Brownstripe Red Snapper Prepared Using Pyloric Caeca and Commercial Proteases. Process Biochem..

[85] Fujita H., Yokoyama K., Yoshikawa M. (2000). Classification and Antihypertensive Activity of Angiotensin I-Converting Enzyme Inhibitory Peptides Derived from Food Proteins. J. Food Sci..

[86] Fujita H., Yamagami T., Ohshima K. (2001). Effects of an Ace-Inhibitory Agent, Katsuobushi Oligopeptide, in the Spontaneously Hypertensive Rat and in Borderline and Mildly Hypertensive Subjects. Nutr. Res..

[87] Forghani B., Zarei M., Ebrahimpour A., Philip R., Bakar J., Abdul Hamid A., Saari N. (2016). Purification and Characterization of Angiotensin Converting Enzyme-Inhibitory Peptides Derived from *Stichopus Horrens*: Stability Study against the ACE and Inhibition Kinetics. J. Funct. Foods.

[88] Ono S., Hosokawa M., Miyashita K., Takahashi K. (2006). Inhibition Properties of Dipeptides from Salmon Muscle Hydrolysate on Angiotensin I-Converting Enzyme. Int. J. Food Sci. Technol..

