# Supplementary material for: Novel Peptide Sequences with ACE-Inhibitory and Antioxidant Activities Derived from the Heads and Bones of Hybrid Groupers (Epinephelus lanceolatus × Epinephelus fuscoguttatus)
Source: Foods. 2022 Dec 9;11(24):3991. doi: 10.3390/foods11243991 (PMC9777584; doi:10.3390/foods11243991)
Supplement: Supplementary file 1 [file foods-11-03991-s001.zip › foods-2017254-supplementary.pdf]

Supplementary Material

Table S1: List of peptide sequences with potential ACE-inhibitory and antioxidant activities identified in HB-IV fraction.

Identified peptide sequences*	Observed mass (Da)	Peptide sequences reported in BIOPEP database#	Identified peptide sequences*	Observed mass (Da)	Peptide sequences reported in BIOPEP database#	Identified peptide sequences*	Observed mass (Da)	Peptide sequences reported in BIOPEP database#
LRL	300.28	RL; LR	MLLP	472.27	LLP	MPVE	474.21	VE
LLAL	428.30	LA	DLDLR	630.33	LR	LGLL	414.28	LG; GL
VAAL	372.24	VAA; AA	VFLAK	576.36	VF; LA	LGFL	448.27	GF; LG
LVVL	442.32	VVL	LDDLR	630.33	LR	TLPF	476.26	LPF
FVSY	514.24	SY	LAGVL	471.31	AG; LA; GV	GPAGPL	510.28	GPL; PL; GPA; AG; GP; AGP
VVGL	386.25	VG; GL	MEPEP	601.24	ME	MLGGPV	572.30	GP; GG; LG; GPV
AAAL	344.21	AA	MPDLR	630.32	LR	DLDGPD	630.25	GP; DG
LGGL	358.22	LG; GL; GG	ETRLL	630.37	RL	LSFL	478.28	SF
LAAL	386.25	AA; LAA; LA	LASVL	501.32	LA	LDAGF	521.25	GF; AG; DA
LGAL	372.24	LG; GA	FADTF	599.26	TF	VAFN	449.23	AF; VAF
AVVL	400.27	AV; AVV; VVL	NNDLR	630.31	LR	LYLF	554.31	YL; LF; LY
LATL	416.26	LA	LLTLR	614.41	LR	LGSF	422.22	SF; LG; GS
LGVL	400.27	LG; GV; LGV	VGSPL	471.27	PL; VG; GS	GLVL	400.27	LVL; GL
LDAL	430.24	DA	KTEAT	548.28	EA; TE	TDAL	418.21	DA
AGPL	356.21	GPL; GP; PL; AG; AGP	SDGNQ	519.19	DG	EASL	418.21	EA; ASL
PCLN	445.20	LN	NLLPA	526.31	LLP	AVGV	344.21	GV; AV; VG
AGVL	358.22	GV; AG	LTAGL	473.28	GL; AG	GVGH	368.18	GV; GH; VG
LLGL	414.28	GL; LG	MGGVF	509.23	VF; GV; MG; GG	AVGL	358.22	VG; GL; AV

Table S1 (*continued*)

Identified peptide sequences*	Observed mass (Da)	Peptide sequences reported in BIOPEP database#	Identified peptide sequences*	Observed mass (Da)	Peptide sequences reported in BIOPEP database#	Identified peptide sequences*	Observed mass (Da)	Peptide sequences reported in BIOPEP database#
FLGM	466.23	LG; GM	LEAL	444.26	EA	LVRGPR	696.44	LVR; PR; GP; VR; RG; VRGP
LGDL	416.23	LG; GD	FDLK	521.28	LK	LVGRPR	696.44	GRP; PR; RP; VG; GR
VGLL	400.27	GL; VG	FCVH	504.22	FC	HLSAFT	674.34	AF; HL
AFLK	477.30	AFL; AF	LPML	472.27	LPM	GPAKSP	555.30	GPA; GP
DPLL	456.26	PL	LSVLK	558.37	LK; SVL	LVCGHL	640.34	GH; HL
EVVE	474.23	EV; VE	DLVDLK	701.40	LK	EVLGVL	628.38	GV; LG; EV; LGV
LGME	448.20	LG; GM; ME	FELNVT	721.36	EL	EDTLAKL	788.43	LA; KL
LGPL	398.25	GPL; PL; GP; LG; LGP	LEAK	459.27	EAK; EA	KDLVDKL	829.49	KL
LNFL	505.29	LNF; NF; LN	LACH	442.20	LA; LAC	VMAPDAR	758.37	DA
VALF	448.27	LF	LKLF	519.34	LK; LF; KL; LKL	YPGLADMR	921.44	YP; PGL; LA; GL; PG; YPG; DM
ALFL	462.28	LF	ELAY	494.24	AY; LAY; LA; EL	PPACLTAL	784.42	PP
EGLM	448.20	EG; GL	MMVF	542.22	MM; VF	DLSNADLR	902.45	LR
DLVE	474.23	VE; LVE	YLDLK	650.36	YL; LK	TEANGPAVT	858.41	GPA; GP; EA; NG; TE; AV
TDYLMK	769.37	TDY; YL, DY	TALALK	615.40	LA; LK	LAFL	463.28	LA; AF; AFL

Table S1 (*continued*)

Identified peptide sequences*	Observed mass (Da)	Peptide sequences reported in BIOPEP database#	Identified peptide sequences*	Observed mass (Da)	Peptide sequences reported in BIOPEP database#	Identified peptide sequences*	Observed mass (Da)	Peptide sequences reported in BIOPEP database#
YPGLWMR	921.45	LW; YP; PGL; GL; PG; YPG; WM; LWM	HGVFK	586.32	VFK; GV; HG; VF	VAVL	400.27	AVL; AV; VAV
VLRL	499.35	LR; RL	GMSGK	478.22	GM; GK; SG	LFDF	540.26	LF; DF
LAVH	438.26	LA; AV	MPLLM	619.31	PL	FAGL	406.22	AG; GL

* Amino acids are designated using their one-letter codes.

BIOPEP database (Minkiewicz *et al.*, 2019) (accessed on July 2021)
